# Supplementary material for: Comparative genomics provides new insights into the diversity, physiology, and sexuality of the only industrially exploited tremellomycete: Phaffia rhodozyma
Source: BMC Genomics. 2016 Nov 9;17:901. doi: 10.1186/s12864-016-3244-7 (PMC5103461; doi:10.1186/s12864-016-3244-7)
Supplement: Additional file 6: — List of orphan genes with links to PFAM (related to Additional file 1: Table S1). (ZIP 1428 kb) [file 12864_2016_3244_MOESM6_ESM.zip › BLAST_HTML_FTR/G00010_P.html]

BLAST Search Results


```
BLASTP 2.2.27+


Reference:
Stephen F. Altschul, Thomas L. Madden, Alejandro A. Schäffer,
Jinghui Zhang, Zheng Zhang, Webb Miller, and David J. Lipman (1997),
"Gapped BLAST and PSI-BLAST: a new generation of protein database
search programs", Nucleic Acids Res. 25:3389-3402.


Reference for
composition-based statistics:
Alejandro A. Schäffer, L. Aravind, Thomas L. Madden, Sergei
Shavirin, John L. Spouge, Yuri I. Wolf, Eugene V. Koonin, and
Stephen F. Altschul (2001), "Improving the accuracy of PSI-BLAST
protein database searches with composition-based statistics and
other refinements", Nucleic Acids Res. 29:2994-3005.


Database: nr
           71,551,133 sequences; 26,053,659,533 total letters


Query= G00010_P

Length=984
                                                                      Score     E
Sequences producing significant alignments:                          (Bits)  Value

emb|CED84348.1|  hypothetical protein [Xanthophyllomyces dendrorh...  1594    0.0  
ref|XP_007310243.1|  hypothetical protein STEHIDRAFT_142670 [Ster...  85.5    2e-13
ref|XP_007262215.1|  hypothetical protein FOMMEDRAFT_144260 [Fomi...  82.4    2e-12
gb|KIP08697.1|  hypothetical protein PHLGIDRAFT_126881 [Phlebiops...  75.1    3e-10
gb|KIK36074.1|  hypothetical protein CY34DRAFT_544303 [Suillus lu...  74.3    6e-10
gb|KDR69868.1|  hypothetical protein GALMADRAFT_214931 [Galerina ...  73.6    9e-10
emb|CEL52581.1|  hypothetical protein RSOLAG1IB_05786 [Rhizoctoni...  67.4    8e-08
gb|KIO34012.1|  hypothetical protein M407DRAFT_17267 [Tulasnella ...  64.7    5e-07
gb|ELU41967.1|  hypothetical protein AG1IA_04003 [Rhizoctonia sol...  63.2    1e-06
gb|KII95436.1|  hypothetical protein PLICRDRAFT_34291 [Plicaturop...  61.2    5e-06
gb|KIM86033.1|  hypothetical protein PILCRDRAFT_86708 [Piloderma ...  60.5    9e-06
gb|KIR84382.1|  hypothetical protein I308_05397 [Cryptococcus gat...  60.5    1e-05
gb|KIJ63498.1|  hypothetical protein HYDPIDRAFT_29304 [Hydnomerul...  59.3    2e-05
gb|KDQ28459.1|  hypothetical protein PLEOSDRAFT_173647 [Pleurotus...  58.9    2e-05
ref|XP_001829909.2|  hypothetical protein CC1G_04598 [Coprinopsis...  56.6    1e-04
gb|EMD32918.1|  hypothetical protein CERSUDRAFT_118352 [Ceriporio...  56.2    2e-04
gb|KEP52424.1|  hypothetical protein V565_045870 [Rhizoctonia sol...  55.5    3e-04
gb|EUC57531.1|  hypothetical protein RSOL_224210 [Rhizoctonia sol...  55.5    3e-04
gb|KLO05862.1|  hypothetical protein SCHPADRAFT_910743 [Schizopor...  53.9    8e-04
ref|XP_007316347.1|  hypothetical protein SERLADRAFT_355167 [Serp...  53.9    0.001
ref|XP_007398245.1|  hypothetical protein PHACADRAFT_259980 [Phan...  53.1    0.001
ref|XP_006460529.1|  hypothetical protein AGABI2DRAFT_220564, par...  53.1    0.001
emb|CDO72665.1|  hypothetical protein BN946_scf184985.g84 [Tramet...  52.4    0.003
ref|XP_003197441.1|  hypothetical protein CGB_N0490C [Cryptococcu...  52.4    0.003
gb|KIY32732.1|  hypothetical protein I305_04890 [Cryptococcus gat...  52.4    0.003
gb|KIM36812.1|  hypothetical protein M413DRAFT_284939 [Hebeloma c...  52.0    0.003
gb|KNZ74333.1|  hypothetical protein J132_07190 [Termitomyces sp....  52.0    0.003
gb|KJA20484.1|  hypothetical protein HYPSUDRAFT_815010 [Hypholoma...  51.6    0.004
emb|CCA69699.1|  hypothetical protein PIIN_03640 [Piriformospora ...  50.8    0.008
gb|KDQ56472.1|  hypothetical protein JAAARDRAFT_180011 [Jaapia ar...  50.8    0.008
gb|KIO10281.1|  hypothetical protein M404DRAFT_21257 [Pisolithus ...  50.4    0.010
gb|KDQ13962.1|  hypothetical protein BOTBODRAFT_132833 [Botryobas...  49.7    0.019
gb|EKC97870.1|  hypothetical protein A1Q2_07873 [Trichosporon asa...  48.1    0.055
gb|KIL61970.1|  hypothetical protein M378DRAFT_25846 [Amanita mus...  48.1    0.060
gb|KIJ13534.1|  hypothetical protein PAXINDRAFT_170416 [Paxillus ...  48.1    0.064
gb|KIR70537.1|  hypothetical protein I310_05787 [Cryptococcus gat...  47.8    0.065
gb|KIR32149.1|  hypothetical protein I352_05381 [Cryptococcus gat...  47.8    0.075
ref|XP_007769210.1|  hypothetical protein CONPUDRAFT_165798 [Coni...  47.8    0.081
ref|XP_007386122.1|  hypothetical protein PUNSTDRAFT_127338 [Punc...  47.0    0.12 
ref|XP_001881514.1|  ABC domain-containing protein [Laccaria bico...  45.4    0.38 
ref|XP_007850649.1|  abc domain-containing protein [Moniliophthor...  45.1    0.45 
gb|EPS94938.1|  hypothetical protein FOMPIDRAFT_1169167 [Fomitops...  44.7    0.55 
gb|KIK25902.1|  hypothetical protein PISMIDRAFT_28753 [Pisolithus...  44.7    0.64 
ref|XP_007866498.1|  hypothetical protein GLOTRDRAFT_121520 [Gloe...  44.3    0.81 
gb|KIM55442.1|  hypothetical protein SCLCIDRAFT_1221129 [Sclerode...  44.3    0.87 
gb|KIR52971.1|  hypothetical protein I315_04429 [Cryptococcus gat...  43.9    1.1  
ref|XP_009553209.1|  hypothetical protein HETIRDRAFT_461425 [Hete...  43.5    1.3  
gb|KIK82139.1|  hypothetical protein PAXRUDRAFT_832381 [Paxillus ...  42.7    2.6  
ref|XP_005383497.1|  PREDICTED: receptor-transporting protein 3 [...  42.0    3.3  


 >emb|CED84348.1| hypothetical protein [Xanthophyllomyces dendrorhous]
Length=809

 Score = 1594 bits (4128),  Expect = 0.0, Method: Compositional matrix adjust.
 Identities = 809/855 (95%), Positives = 809/855 (95%), Gaps = 46/855 (5%)

Query  129  MESDLDTDDYRSQDSPHEFPSNQLIEPNQVRALMDIFPPQVDERSSGEVISEHRLLELQS  188
            MESDLDTDDYRSQDSPHEFPSNQLIEPNQVRALMDIFPPQVDERSSGEVISEHRLLELQS
Sbjct  1    MESDLDTDDYRSQDSPHEFPSNQLIEPNQVRALMDIFPPQVDERSSGEVISEHRLLELQS  60

Query  189  RRLASEHRRKLLARVPAGGRNGKQWKASDLPLKIIRQIIHLVAISIVTEEVQAKLADGDN  248
            RRLASEHRRKLLARVPAGGRNGKQWKASDLPLKIIRQIIHLVAISIVTEEVQAKLADGDN
Sbjct  61   RRLASEHRRKLLARVPAGGRNGKQWKASDLPLKIIRQIIHLVAISIVTEEVQAKLADGDN  120

Query  249  RIHFGKRPVGLTIAHIRARSRIAPLRLVCRAWKQATETIPFWSELATVFGFPHNIRPYRE  308
            RIHFGKRPVGLTIAHIRARSRIAPLRLVCRAWKQATETIPFWSELATVFGFPHNIRPYRE
Sbjct  121  RIHFGKRPVGLTIAHIRARSRIAPLRLVCRAWKQATETIPFWSELATVFGFPHNIRPYRE  180

Query  309  NDCLITQLCWVCHFARFPLSQPAAYVRTTLGTVWICKLHLAVPFCGRCLRSRIDPAFRSR  368
            NDCLITQLCWVCHFARFPLSQPAAYVRTTLGTVWICKLHLAVPFCGRCLRSRIDPAFRSR
Sbjct  181  NDCLITQLCWVCHFARFPLSQPAAYVRTTLGTVWICKLHLAVPFCGRCLRSRIDPAFRSR  240

Query  369  RLQWIGPRSSLPEYTAPNEDIDPGLRLTEDDDVEDEDDDINGGVNAGRELYGPNMACCQR  428
            RLQWIGPRSSLPEYTAPNEDIDPGLRLTEDDDVEDEDDDINGGVNAGRELYGPNMACCQR
Sbjct  241  RLQWIGPRSSLPEYTAPNEDIDPGLRLTEDDDVEDEDDDINGGVNAGRELYGPNMACCQR  300

Query  429  CRYECLQEAVTKRDPAVRIDLEDNGDLECFFQYVERAQGSAESIAVDLLERRWLARWTKF  488
            CRYECLQEAVTKRDPAVRIDLEDNGDLECFFQYVERAQGSAESIAVDLLERRWLARWTKF
Sbjct  301  CRYECLQEAVTKRDPAVRIDLEDNGDLECFFQYVERAQGSAESIAVDLLERRWLARWTKF  360

Query  489  HDLATQVVQTEKINPVTAASADGNDMRAIFKASMGIEGDGSEPMTEALERELAQFEYEAA  548
            HDLATQVVQTEKINPVTAASADGNDMRAIFKASMGIEGDGSEPMTEALERELAQFEYEAA
Sbjct  361  HDLATQVVQTEKINPVTAASADGNDMRAIFKASMGIEGDGSEPMTEALERELAQFEYEAA  420

Query  549  VAREENAARVVGAEHNNSDGERQTDEEGQGQEDEEEEEEEEEEEGVELTPGGYIQIVEMA  608
            VAREENAAR                                              IVEMA
Sbjct  421  VAREENAAR----------------------------------------------IVEMA  434

Query  609  TEAWIHQRVIDGFWVSPLEQRAFQETFSQQTPSSAEGDITLDVVHPLSSSTLGSSVCTTS  668
            TEAWIHQRVIDGFWVSPLEQRAFQETFSQQTPSSAEGDITLDVVHPLSSSTLGSSVCTTS
Sbjct  435  TEAWIHQRVIDGFWVSPLEQRAFQETFSQQTPSSAEGDITLDVVHPLSSSTLGSSVCTTS  494

Query  669  SHPNGPLPPAGGALDQKTINLYSRKLRSILLPGLGQIARLCALQKDPLNVAPKLGIHKLL  728
            SHPNGPLPPAGGALDQKTINLYSRKLRSILLPGLGQIARLCALQKDPLNVAPKLGIHKLL
Sbjct  495  SHPNGPLPPAGGALDQKTINLYSRKLRSILLPGLGQIARLCALQKDPLNVAPKLGIHKLL  554

Query  729  ELLRLPGAWVHGGLDCMELALDQADGPEQISTMPAEILSPPPLIRHPNNISPSESLSFMP  788
            ELLRLPGAWVHGGLDCMELALDQADGPEQISTMPAEILSPPPLIRHPNNISPSESLSFMP
Sbjct  555  ELLRLPGAWVHGGLDCMELALDQADGPEQISTMPAEILSPPPLIRHPNNISPSESLSFMP  614

Query  789  TSSTAKMYTIESPSSTDSISPSAPSSHTTSPIRTGSSPPTSLSPPSCAPTIALITPDPPI  848
            TSSTAKMYTIESPSSTDSISPSAPSSHTTSPIRTGSSPPTSLSPPSCAPTIALITPDPPI
Sbjct  615  TSSTAKMYTIESPSSTDSISPSAPSSHTTSPIRTGSSPPTSLSPPSCAPTIALITPDPPI  674

Query  849  VSSTLSSVPSPKPNPAQNMKKIDPRFPLSPKTSRPVPQIPRVPLDASRLGLYTKDIIKRA  908
            VSSTLSSVPSPKPNPAQNMKKIDPRFPLSPKTSRPVPQIPRVPLDASRLGLYTKDIIKRA
Sbjct  675  VSSTLSSVPSPKPNPAQNMKKIDPRFPLSPKTSRPVPQIPRVPLDASRLGLYTKDIIKRA  734

Query  909  WWDACDDMRRCVCSICLRGLKAQREGRQDEGIVSDREPKDENDEMQEEQEERGKRKGGNE  968
            WWDACDDMRRCVCSICLRGLKAQREGRQDEGIVSDREPKDENDEMQEEQEERGKRKGGNE
Sbjct  735  WWDACDDMRRCVCSICLRGLKAQREGRQDEGIVSDREPKDENDEMQEEQEERGKRKGGNE  794

Query  969  DSRAGDKKQRIEVDD  983
            DSRAGDKKQRIEVDD
Sbjct  795  DSRAGDKKQRIEVDD  809


>ref|XP_007310243.1| hypothetical protein STEHIDRAFT_142670 [Stereum hirsutum FP-91666 
SS1]
 gb|EIM80774.1| hypothetical protein STEHIDRAFT_142670 [Stereum hirsutum FP-91666 
SS1]
Length=986

 Score = 85.5 bits (210),  Expect = 2e-13, Method: Compositional matrix adjust.
 Identities = 153/714 (21%), Positives = 256/714 (36%), Gaps = 219/714 (31%)

Query  259  LTIAHIRARSRIAPLRLVCRAWKQATETIPFWSELATVF---------------GFPHNI  303
            L IA +R +  +  L  VC +W+ A E   FW      F               G  + +
Sbjct  74   LYIA-LRDQVYVEKLMKVCPSWQAALEQHTFWQHAIYAFDPADILINTSTIVQTGSSNTV  132

Query  304  R--PYRENDCLITQLCWVCHFARFPLSQPAAYV------RTTL----GTVWICKLHLAVP  351
            R  PYR    ++T  C VC      L+ P +Y+      R  L    G V +CK H    
Sbjct  133  RVSPYRHFRHIVTHSCTVCR-----LNTPLSYLGLNNAKRFVLLPYHGHVSVCKEHHKRD  187

Query  352  FCGRCLRSRIDPAFRSRRLQWIGPRSSLPEYTAPNEDIDPGLRLTEDDDVEDEDDDINGG  411
            FCG CLRS   P              S  ++ A +E I       E++D+E         
Sbjct  188  FCGVCLRSPCPPHL------------SYVQHQAESEMIA----CAENEDLET--------  223

Query  412  VNAGRELYGPNM-ACCQRCRYECL----------QEAVTKRDPAVRIDLEDNGDLECFFQ  460
                     PN+   C+ CR E L          +EAV +  P    D  D    +    
Sbjct  224  --------WPNIFTTCKYCRSEALWKRMSNSPADREAVGQGPPQWAAD--DWETRQVIDT  273

Query  461  YVERAQGSAESIAVDLLERRWLARWTKFHDLATQVVQTEKINPVTAASADGNDMRAIFKA  520
            +++  +G+ + +     E+ WL R+T+F D+                             
Sbjct  274  FIDLGEGTVDEVIRVAQEKNWLRRYTRFADI-----------------------------  304

Query  521  SMGIEGDGSEPMTEALERELAQFEYEAAVAREENAARVVGAEHNNSDGERQTDEEGQGQE  580
                           +E+ +A  +YE  +         V      ++GE   D+  + ++
Sbjct  305  ---------------MEQAVASQKYELKLTGGGAGGGYV------NEGE---DDSFELED  340

Query  581  DEEEEEEEEEEEGVELTPGGYIQIVEMATEAWIHQRVIDGFWVSPLEQRAFQETFSQQTP  640
            D  E  +  E+ G +            A EAW+ +R++DG W+SP +    Q   ++  P
Sbjct  341  DSFELHQRAEDSGAKAP----------ACEAWVRERILDGHWISPADDYYNQRPAAELLP  390

Query  641  SSAEGDITLDVVHPLSSSTLGSSVCTTSSHPNGPLPPAGGA--LDQKTINLYSRKLRSIL  698
            ++          HP +      +V T    P     PA     L +    ++  K R IL
Sbjct  391  AAE---------HPAAWLLDEHAVTTRHPTPKTIAVPAAPTYHLAEALYRIHKVKTREIL  441

Query  699  LPGLGQIARLCALQ----KDPLNVAPKLGIHKLLELLRLPGAWVHGGLDCMELALDQADG  754
            LP +  I +   L     ++ +  A KL + +L ++LR    WV                
Sbjct  442  LPPMSNIVKRIMLDSRNGEEAIRRASKLNLEELFQVLRETWPWVK---------------  486

Query  755  PEQISTMPAEILSPPPLIRHPNNISPSESLSFMPTSSTAKMYTIESPSSTDSISPSAPSS  814
             EQ++    E +    +++H ++ S S + S    +++  + T                 
Sbjct  487  -EQLAD---EHVRKAAVLKHADDSSSSSTKSDGSHTTSPVLST-----------------  525

Query  815  HTTSPIRTGSSPPTSLSPPSCAPTIALITPDPPIVSSTLSSVPSPKPNPAQNMKKIDPRF  874
               + ++T  SPP   + P+   T+ +I P P  ++S            A ++ +  P  
Sbjct  526  ---TTLQTTPSPPPKDAGPASDDTLDVIGPRPDEMASMCME--------ASSLMRYIPFV  574

Query  875  PLSPKTSRPVPQIPRVPLDASRLGLYTKDIIKRAWWDACDDMRRCVCSICLRGL  928
            PL+                A  L  Y+ + IK  W +AC  +  C CSIC R L
Sbjct  575  PLT----------------AECLPPYSHEAIKSVWREACAPLYHCACSICKRTL  612


>ref|XP_007262215.1| hypothetical protein FOMMEDRAFT_144260 [Fomitiporia mediterranea 
MF3/22]
 gb|EJD08263.1| hypothetical protein FOMMEDRAFT_144260 [Fomitiporia mediterranea 
MF3/22]
Length=1315

 Score = 82.4 bits (202),  Expect = 2e-12, Method: Compositional matrix adjust.
 Identities = 174/816 (21%), Positives = 281/816 (34%), Gaps = 227/816 (28%)

Query  201   ARVPAGGRNGKQWKASDLPLKIIRQII--HLVAISIVTEEVQAKLADGDNRIHFGKRPVG  258
             A V  G    + W  + LP ++IR I   +L+A+S  T  + A     +N       P  
Sbjct  515   AMVYKGKSTTRSW--ATLPPEVIRLITTHYLLAMS-ATAVLPAVWQQRENW------PGR  565

Query  259   LTIAHIRARSRIAPLRLVCRAWKQATETIPFWSELATV---------FGFPHNIRPYR--  307
             +    +R    I  L  V  AW  A E   FW + A V         F     + PYR  
Sbjct  566   MAFTVVRDAEVIQQLMRVSPAWASAIEYHLFWQQAAAVLDAADHVLAFARAQRVSPYRHF  625

Query  308   -----ENDCLITQLCWVCHFARFPLSQPAAYVRTTLGTVWICKLHLAVPFCGRCLRSRID  362
                  +  C+  ++ +    +   +++   Y  T LG V  C+ H    FCG CLR    
Sbjct  626   RQHLFQRSCIPCRINYPNSTSGLLIARKLTYTHT-LGFVPCCREHRKERFCGICLRD---  681

Query  363   PAFRSRRLQWIGPRSSLPEYTAPNEDIDPGLRLTEDDDVEDEDDDINGGVNAGRELYGPN  422
                                             L E+   E+E+ D    V A        
Sbjct  682   ------------------------------ASLDENCIAENEECDYWQSVEA--------  703

Query  423   MACCQRCRYECLQEA-----------VTKRDPAV---RIDLEDNGDLECFFQYVERAQGS  468
                C+ CR ECL +A           +TK   AV       ED    +    +VE  +GS
Sbjct  704   --TCRTCRAECLWKACCEGQEQPGNRLTKEAEAVGGQNFAPEDWEARQTIDAFVEMGEGS  761

Query  469   AESIAVDLLERRWLARWTKFHDLATQVVQTEKINPVTAASADGNDMRAIFKASMGIEGDG  528
                +    +E+RWL ++TK  ++    V T +I                           
Sbjct  762   VRDVIGLCMEKRWLRKYTKIAEMMRLAVATSRI---------------------------  794

Query  529   SEPMTEALERELAQFEYEAAVAREENAARVVGAEHNNSDGERQTDEEGQGQEDEEEEEEE  588
                              ++ +    N A ++G+     + + +  E  +  ED E     
Sbjct  795   -----------------QSRMVENANEAGILGSGMGEYEDDEELSEVDEDDEDPELLSIT  837

Query  589   EEEEGVELTPGGYIQIVEMATEAWIHQRVIDGFWVSPLEQRAFQETFSQQTPSSAEGDIT  648
             EE +GV           E+A   W   R++DG W SP +Q      +  Q P+++     
Sbjct  838   EETQGVR----------ELAVNDWARARILDGHWYSPADQ-----WYLIQGPNNSNAQRL  882

Query  649   -----------LDVVHPLSSSTLGSSVCTTSSHPN----GPLPPAGGALDQKTINLYSRK  693
                        +  +HP+S + + S       HP+       PP   +L     + Y R+
Sbjct  883   STNQPYPPVPYIPAIHPVSWTVISS--VAERPHPDPESVRSSPPPSLSLCGAAFDAYRRQ  940

Query  694   LRSILLPGLGQIARL----CALQKDPLNV-APKLGIHKLLELLRLPGAWVHGGLDCMELA  748
             +R++L P +  I R     CA+     +V   ++ I  +   LR    W +G        
Sbjct  941   MRAVLFPAMANIVRKVVMECAVDGTDASVRVARMSIEDVANALRDDMTWYNG--------  992

Query  749   LDQADGPEQISTMPAEILSPPPLIRHPNNISPSESLSFM------PTSSTAKMYTIESPS  802
             +D AD   +     ++  S           SP  S S +      P +S  K+ + E  +
Sbjct  993   VDWADRRRRREVNSSDDSSSGSEKSGSGTTSPVLSTSTLQTTPSPPPASDVKLQSSEQTN  1052

Query  803   STDSISPSAPSSHTTSPIRTGSSPPTSLSPPSCAPTIALITPDPPIVSSTLSSVPSPKPN  862
             S     PS+ SSH+  P+    S P ++SP   +PT     P  P +  ++S +PS    
Sbjct  1053  SMPPPPPSSSSSHSRQPLSPIMSLPIAISPVLESPT---QIPSIPFIPESMSEMPS----  1105

Query  863   PAQNMKKIDPRFPLSPKTSRPVPQIPRVPLDASRLGLYTKDIIKRAWWDACDDMRRCVCS  922
                                                  +T +  K  W DAC  + +C C 
Sbjct  1106  -------------------------------------FTTETFKNIWRDACAPLFQCRCK  1128

Query  923   ICLRG-LKAQREGRQDEGIVSDREPKDENDEMQEEQ  957
             IC R  L+A  E      + S   P  ++ E  ++Q
Sbjct  1129  ICERAVLRANIEA--GNIVPSQHYPHTQDQEYAQKQ  1162


>gb|KIP08697.1| hypothetical protein PHLGIDRAFT_126881 [Phlebiopsis gigantea 
11061_1 CR5-6]
Length=815

 Score = 75.1 bits (183),  Expect = 3e-10, Method: Compositional matrix adjust.
 Identities = 184/819 (22%), Positives = 285/819 (35%), Gaps = 248/819 (30%)

Query  204  PAG--GRNGKQWKASDLPLKIIRQI-IHLVAISIVTEEVQAKLADGDNRIHFGKRPVGLT  260
            P+G  G+  + W  + LP +IIR +  HL+AI  VT ++       + +  +  R   + 
Sbjct  27   PSGYKGKVTRSW--AQLPPEIIRLVATHLLAI--VTPQLYLP-GIWEYKDFWHSR---IV  78

Query  261  IAHIRARSRIAPLRLVCRAWKQATETIPFWSE-LATVFGFPH------------------  301
               +R    +  L  VC  W+ A E  PFW    A +    H                  
Sbjct  79   YTALRDAMEVERLMGVCVPWQSALEYHPFWHHACAAIDPTDHLAQHAMIRPPASAGSNKA  138

Query  302  ---NIRPYRENDCLITQLCWVCHFARFP-----LSQPAAYVRTTLGTVWI-CKLHLAVPF  352
                I P++    + T  C VC    FP     L     +V +   T++  C+ H    F
Sbjct  139  SLLRISPHKHFRQMSTYSCMVCRL-NFPTTLAGLCSGKRFVYSPQLTMFTACRDHRKGTF  197

Query  353  CGRCLRSRIDPAFRSRRLQWIGPRSSLPEYTAPNEDIDPGLRLTEDDDVEDEDDDINGGV  412
            C  CLR+                        AP++D    + +      E+ED++   GV
Sbjct  198  CSLCLRA------------------------APHDDTHLMVCI-----AENEDEETWPGV  228

Query  413  NAGRELYGPNMACCQRCRYECLQEAVTKRDPAVRIDLEDNG-------DLECFFQYVERA  465
             A           C+ CR E L      RDP  R  L  +        D E   Q VE  
Sbjct  229  EA----------TCRHCRGEGLWRRAL-RDPLDREALGSHPLKWRHPPDWE-IGQAVETF  276

Query  466  QGSAESIAVDLL----ERRWLARWTKFHDLATQVVQTEKINPVTAASADGNDMRAIFKAS  521
                E    D+L    E+ WL   TK  +L +Q + +                R + +  
Sbjct  277  MDMGEGCIADVLCLTREKHWLRTHTKIAELLSQALASS---------------RYVARTE  321

Query  522  MGIEGDGSEPMTEALERELAQFEYEAAVAREENAARVVGAEHNNSDGERQTDEEGQGQED  581
             G EGD                                            +DE+  G+E+
Sbjct  322  AGYEGD--------------------------------------------SDEDLSGEEE  337

Query  582  EEEEEEEEEEEGVELTPGGYIQIVEMATEAWIHQRVIDGFWVSPLEQRAFQETFSQ----  637
            ++ E     E+      GG   I E+A   W   R++DG WV+P +Q        Q    
Sbjct  338  DDPELMSLTEDA-----GG---IRELAITDWARNRILDGHWVAPADQWYENSVPGQPDVV  389

Query  638  --QTPSSAEGDITLDVVHPLSSSTLGSSVCTTSSHP-----NGPLPPAGGALDQKTINLY  690
              Q P    G      +    S+  G  +     HP       P PP   AL +     Y
Sbjct  390  PAQHPCPWHGATYSGALQDGESTGDGEEL----EHPRPRTYQAPCPPT-FALCETVYRTY  444

Query  691  SRKLRSILLPGLGQIARLCALQK-----DPLNVAPKLGIHKLLELLRLPGAWVHGGLDCM  745
             R +R ILLPG+  I R   ++      DP   A K+ +  +++ LR    W   G+D  
Sbjct  445  QRVMREILLPGMSNIVRRILMESQVDGVDPTIPASKMTLEDVVKELRDHATW-SKGIDWA  503

Query  746  E--LALDQADGPEQISTMPAEILSPPPLIRHPNNISPSESLSFMPTSSTAKMYTIESPSS  803
            E  +   + DG  +  ++ A+  S         +   S S +  P  ST  + T  SP  
Sbjct  504  ERRIIRGRQDGLRRQRSVDADSTS---------SSRSSGSHTTSPVLSTTTLQTTPSP--  552

Query  804  TDSISPSAPSSHTTSPIRTGSSPPTSLSPPSCAPTIALITPDPPIVSSTLSSVPSPKPNP  863
                                  PP+S+     AP+       P  VSS + ++  P   P
Sbjct  553  ----------------------PPSSVKDEELAPS-------PVAVSSPIMAILEP---P  580

Query  864  A-QNMKKIDPRFPLSPKTSRPVPQIPRVPLDASRLGLYTKDIIKRAWWDACDDMRRCVCS  922
            A Q  + I P              IP VP+  + L  Y+ + +K  W +AC  + +C CS
Sbjct  581  ALQTHELIRP--------------IPYVPMTIAHLPPYSMECLKSIWREACAPLYQCRCS  626

Query  923  ICLRGLKAQREGRQDEGIVSDREPKDENDEMQEEQEERG  961
            IC R + A         +     P+ + D++  +Q  + 
Sbjct  627  ICERAMLAA-------NVNPKNTPQKQQDQVSADQARKA  658


>gb|KIK36074.1| hypothetical protein CY34DRAFT_544303 [Suillus luteus UH-Slu-Lm8-n1]
Length=949

 Score = 74.3 bits (181),  Expect = 6e-10, Method: Compositional matrix adjust.
 Identities = 158/728 (22%), Positives = 247/728 (34%), Gaps = 197/728 (27%)

Query  300  PH-NIRPYRENDCLITQLCWVCHFARFPLSQPAAYVRTTLGTVWICKLHLAVPFCGRCLR  358
            PH + R    + C+I ++   C       ++   +V   LG + +C+ H  + FCG CLR
Sbjct  75   PHTHFRTMTYHSCIICRINSPCSSVGLGAAK-RTHVIPRLGVISVCREHRKITFCGVCLR  133

Query  359  ----SRIDPAF---RSRRLQWIGPRSS-------LPEYTAPNEDIDPGLRLTEDDDVEDE  404
                +  DP F    + RL       S       L  Y      ++P L +      E+E
Sbjct  134  EAPAAECDPQFDIQSNMRLSGYNQLYSHGSFNLALGGYNPYATTLNPALMVLCS---ENE  190

Query  405  DDDINGGVNAGRELYGPNMACCQRCRYECL----------QEAV----------------  438
            DDD   GV+A           C+ CR E L          +EA+                
Sbjct  191  DDDTWPGVDA----------TCRSCRSEGLWNRIKASARDREAIGGPRFVQSLSSSRSSP  240

Query  439  -------TKRDPAVRIDLEDNGDLECFFQYVERAQGSAESIAVDLLERRWLARWTKFHDL  491
                     R P  +    D    +    ++E  +GS   +     E+ WL  +TK  +L
Sbjct  241  ASPRQLALARVPPWKYASADWETRQTVDAFIELGEGSITEVLNVAREKLWLRSYTKMPEL  300

Query  492  ATQVVQTEKINPVTAASADGNDMRAIFKASMGIEGDGSEPMTEALERELAQFEYEAAVAR  551
              Q V   +                   AS G+  D S+             E E A A 
Sbjct  301  MEQAVAATRW------------------ASEGMGTDFSQ-------NSYHHREPETAAAP  335

Query  552  EENAARVVGAEHNNS--------DGERQTDEEGQGQEDEEEEEEEEEEEGVELT--PGGY  601
             ++  R      + S           R          D E  E+E++ + + +T   GG 
Sbjct  336  PQDHTRTTEGGDSYSPVNSMSPSRRSRSPSPRSAYSSDYEGSEDEDDPDLLSMTEDAGG-  394

Query  602  IQIVEMATEAWIHQRVIDGFWVSPLEQRAF-------------------QETFSQQTPSS  642
              + ++A   W   R++DG W SP +Q                       ET   +   S
Sbjct  395  --VRDLAIADWARTRILDGHWCSPADQWYGYARPIGGGVRDPGEEPDYESETDDDEPLES  452

Query  643  AEGD--ITLDVVHPL------SSSTLGSSVCTTSSHPN-----GPLPPAGGALDQKTINL  689
             +    + +   HP         S       T  SHP         PP+ G  +Q     
Sbjct  453  GQSHRRVRVPATHPCPWTLSRPPSPSSLPSPTDDSHPRISTVRSQAPPSFGLCEQ-AFRA  511

Query  690  YSRKLRSILLPGLGQIARLCALQK---------DPLNVAPKLGIHKLLELLRLPGAWVHG  740
            +S++LR ILLPG+  I R   ++          DP   A K+ +  +L  LR PG W   
Sbjct  512  FSKQLRQILLPGMNNIVRRLVIESTNGIGGAWADPAVRASKMNLEDVLAELRTPGVWF-D  570

Query  741  GLDCMELALDQADGPEQISTMPAEILSPPPLIRHPNNISPSESLSFMPTSSTAKMYTIES  800
            G+D     LD     E  S                                         
Sbjct  571  GVDWAAGGLDIGGKREDDSL----------------------------------------  590

Query  801  PSSTDSISPSAPSSHTTSPIRTGSSPPTSLSPPSCAPTIALITPDPPIVSSTLSSVPSPK  860
              S+ S S  +  SHTTSP+ + ++  T+ SPP   P+ +      P      +++ +  
Sbjct  591  --SSTSASTRSDGSHTTSPVLSTTTLHTTPSPP---PSHSEEKEHEP------ATITARP  639

Query  861  PNPAQNMKKIDPRFPLSPKTSRPVPQIPRVPLDASRLGLYTKDIIKRAWWDACDDMRRCV  920
              P   +         SPK   P   IP VP+  + +  Y+ +  K+ W +AC  +  C 
Sbjct  640  KPPQVVVPIPVSPVLESPKLLHP---IPYVPVTLAHMAQYSLEAFKQVWREACAPLYHCR  696

Query  921  CSICLRGL  928
            CS+C R +
Sbjct  697  CSVCERAM  704


>gb|KDR69868.1| hypothetical protein GALMADRAFT_214931 [Galerina marginata CBS 
339.88]
Length=955

 Score = 73.6 bits (179),  Expect = 9e-10, Method: Compositional matrix adjust.
 Identities = 85/349 (24%), Positives = 146/349 (42%), Gaps = 53/349 (15%)

Query  600  GYIQIVEMATEAWIHQRVIDGFWVSPLEQRAFQETFSQQTPSSAEGDITLDVVHPLSSST  659
            G  Q+ ++A + W  +R++DG W SP +         Q T         +  VHP   S 
Sbjct  339  GSGQVRDLALQDWARKRILDGHWFSPADTWYNHSIPGQPT--------VVPTVHPCPWSR  390

Query  660  LGSSVCTTSSHPNGPLP-------PAGGALDQKTINLYSRKLRSILLPGLGQIARLCALQ  712
                V  ++     P+P       P   AL ++    + +++R IL P L  I R   ++
Sbjct  391  ELEGVDPSAEEERHPVPAIVRGKIPPSFALCEQAYVTHMKQMREILGPALKNIVRKLVME  450

Query  713  ---------KDPLNVAPKLGIHKLLELLRLP-GAWVHGGLDCMELALDQADGPEQISTMP  762
                      DP   A K+ I +++ +LR   G W + G+D +E   ++ +   +  +  
Sbjct  451  CSMMTDKGYDDPAQKARKMHIEEVVTILREEEGVW-YDGVDWIEKKRNEEEALRRRRSEA  509

Query  763  AEILSPPPLIRHPNNISPSESLSFMPTSSTAKMYTIESPSSTDSISPSAP-SSHTTSPIR  821
            AE      + +        +S S   +SS           S +S+  S   SS+ TSP+ 
Sbjct  510  AEAALRNAVAQDTRRRKEDDSSSTTSSSS----------GSNESVKSSVHGSSNATSPVL  559

Query  822  TGSSPPTSLSPPSCAPTIALITPDPPIVSSTLSSVPSPKPNPAQNMKKIDPR-FPLSPKT  880
            + ++  T+ SPP             P+    L+++   +    Q M    PR  P+ P  
Sbjct  560  STTTLQTTPSPP-------------PLSEEGLAALKEEENAERQRMYAAVPRTIPVDPVR  606

Query  881  SRP--VPQIPRVPLDASRLGLYTKDIIKRAWWDACDDMRRCVCSICLRG  927
            S P  + +IP VP+  + +  Y+ + +K AW +AC  +  C CSIC R 
Sbjct  607  STPRLLSKIPYVPVTTAHMPHYSMEALKAAWREACAPLYHCCCSICERA  655


 Score = 64.3 bits (155),  Expect = 6e-07, Method: Compositional matrix adjust.
 Identities = 69/297 (23%), Positives = 112/297 (38%), Gaps = 79/297 (27%)

Query  276  VCRAWKQATETIPFWSELATVFGFPHNI---------RP-----------------YREN  309
            +C  W++A ET  FW++   +   PH+I         +P                 Y+  
Sbjct  80   ICPEWRKAVETHLFWNQAINILD-PHDILLFHMVIHPKPQLNASSNAQQAPIYLTHYQHL  138

Query  310  DCLITQLCWVCHFARFPLSQPA------AYVRTTLGTVWICKLH--LAVPFCGRCLRSRI  361
              ++   C VC    +PLS             T LGTV IC+ H      FCG CLR   
Sbjct  139  RNILNSTCLVCRI-NYPLSNSGLASAKRVITNTMLGTVAICREHDRRRTAFCGLCLRD--  195

Query  362  DPAFRSRRLQWIGPRSSLPEYTAPNEDIDPGLRLTEDDDVEDEDDDINGGVNAGRELYGP  421
                         P +   + T+   +I   L + E+     EDDD             P
Sbjct  196  ------------SPLAHTQDTTSREYEITQSLGIHEN-----EDDDT-----------WP  227

Query  422  NM-ACCQRCRYECLQEAVTKRDPAVRI---------DLEDNGDLECFFQYVERAQGSAES  471
            N+ + C++CR E L  A ++ +  +           D E   ++E F   +E A+G+ + 
Sbjct  228  NVESTCRKCRTEWLWRAASQNEQDIEAIGGTAMSSADWETRQNVEGF---IELAEGAIQD  284

Query  472  IAVDLLERRWLARWTKFHDLATQVVQTEKINPVTAASADGNDMRAIFKASMGIEGDG  528
            +     E+ WL   TK+H L   ++   +  P T A  +  +     +  M + G G
Sbjct  285  VITLAREKYWLRHCTKYHSLGQHLLAAHRTEPRTEAQEEEEEDSEEDRELMLMRGSG  341


>emb|CEL52581.1| hypothetical protein RSOLAG1IB_05786 [Rhizoctonia solani AG-1 
IB]
Length=1212

 Score = 67.4 bits (163),  Expect = 8e-08, Method: Compositional matrix adjust.
 Identities = 74/285 (26%), Positives = 114/285 (40%), Gaps = 68/285 (24%)

Query  702   LGQIARLCALQK---DPLNVAPKLGIHKLLELLRLPGAWVHGG---------------LD  743
             L  I   C L +   DP  V  ++G  +L E+LR P  WV GG                D
Sbjct  841   LESIRSQCVLSRGEPDPCKVVCRIGADRLFEILRGPEPWVEGGGWEDVYVDWEDADSSRD  900

Query  744   CMELA---------------LDQADGPEQI--STMPAEILSPPPLIRHPNNISPSESLSF  786
              M                  L+QA+ P +   S  P+ +    P +R     SP  S + 
Sbjct  901   SMTPGHFRDHGSATPNNFRDLEQANAPREREPSLTPSSVA---PTLRESVAPSPPPS-TI  956

Query  787   MPTSSTAKMYTIESPSSTDSISPSAPSSHTTSPIRTGSSPPTSLSPPSCAPTIALITPDP  846
                S      +  SP S+ +ISP +P+   +SP+ T  SP    SP     +  L TP P
Sbjct  957   SAQSPPPTTISAHSPRSS-TISPPSPTDQRSSPLSTTPSPEALTSP-----STMLTTPSP  1010

Query  847   P-------IVSSTLSSVPSPKPNPAQNMKKI-------------DPRFPLSPKTSRPVPQ  886
             P       +++ T + V   +    +N+  +              PR P SP   +P+  
Sbjct  1011  PPIVGGKKLINETDNYVTHSQ---VENLTHLHTHSQTRTENFIPSPRLPTSPILKKPIQP  1067

Query  887   IPRVPLDASRLGLYTKDIIKRAWWDACDDMRRCVCSICLRGLKAQ  931
             IP +P     LG +TK +++  W +A   +  C C+IC R ++ Q
Sbjct  1068  IPLIPDPTYYLGQHTKAVLEMLWREATGGLWMCRCTICTRAMQVQ  1112


>gb|KIO34012.1| hypothetical protein M407DRAFT_17267 [Tulasnella calospora MUT 
4182]
Length=796

 Score = 64.7 bits (156),  Expect = 5e-07, Method: Compositional matrix adjust.
 Identities = 173/785 (22%), Positives = 278/785 (35%), Gaps = 200/785 (25%)

Query  207  GRNGKQWKASDLPLKIIRQIIHLVAISIVTEEVQAKLADGDNRIHFGKRPVGLTIAHIRA  266
            G+   +   +DLP  ++R + +L+     T           +   +G        A + A
Sbjct  20   GKVATKGSWADLPHDVLRLVANLLFSGTFTSWYSHPTPAWRD---WGAYKQPQLWATLMA  76

Query  267  RSRIAPLRLVCRAWKQATETIPFWSELATVFG--------FPH-----------------  301
               ++PLRLVC  W+ A ET   ++               +PH                 
Sbjct  77   LQDVSPLRLVCMNWQAAMETHQIYNVACNNLDPQYLDAQLYPHIPQLPQPAGGGALNAAF  136

Query  302  --------NIRPYRENDCLITQLCWVCHFARFPLSQPAAYVRTTL-----GTVWICKLHL  348
                      R Y       ++ C  C FA    S+      +T+     G V +C  H 
Sbjct  137  GARRARNTAQRTYTTYARTFSRCCLPCRFASPQDSKGVNIATSTMWSRYFGDVGVCDRHR  196

Query  349  AVPFCGRCLRSRIDPAFRSRRLQWIGPRSSLPEYTAPNEDIDPGLRLTEDDDVEDEDDDI  408
                CG CL+      +R      +        +   N  I              EDDD 
Sbjct  197  PEVVCGYCLKDYYLDCWRFNNHGQL-------YHDQRNRTI-----------AVTEDDDY  238

Query  409  NGGVNAGRELYGPNMACCQRCRYECLQEAVTKRDPAVRIDLEDNGDLECFFQYVERAQGS  468
              G+          +A C+ CR   ++  V  + P    DL D+        ++E  +G+
Sbjct  239  CRGI----------IATCRSCRSVLIRNLVRTKIPQQASDLTDS----VLDTFIECGEGN  284

Query  469  AESIAVDLLERRWLARWTKFHDLATQVVQTEKINPVTAASADGNDMRAIFKASMGIEGDG  528
             + +  ++ E+ WLA  T F +L    +  E++           ++RA  +A   +EG  
Sbjct  285  IKDLLAEVDEKVWLATRTNFRELHDSALAVERMRL-------KQELRAEREAEGIMEGRV  337

Query  529  SEPMTEALERELAQFEYEAAVAREENAARVVGAEHNNSDGERQTDEEGQGQEDEEEEEEE  588
            S   TEA  R  A  +  A+    E+A ++                  Q  E+EE++E  
Sbjct  338  S---TEA--RLQALLDSGASPEEIEDALQM------------------QAVEEEEDDELL  374

Query  589  EEEEGVELTPGGYIQIVEMATEAWIHQRVIDGFWVSPLE-------QRAFQETFSQQTPS  641
            ++E G  LT      + +MA   W   R+++G W+SPL+        R   E   Q+ P 
Sbjct  375  DDEYGEVLTAAEEANVNDMAISGWARNRIMEGCWLSPLDIWHNDGGVRVRTEIL-QRLPQ  433

Query  642  SAEGDITLDVVHPLSSSTLG---------SSVCTTSSHPN-----GPLPPAGGALDQKTI  687
            S          HP++S   G         + +      P       P PPA   + Q+  
Sbjct  434  SW---------HPVNSYLNGPDPMADLHQAPIPQNQMKPRLTSIRCPHPPA--RIFQRAQ  482

Query  688  NLYSRKLRSILLPGLGQIAR--LC---ALQKDPLNVAPKLGIHKLLELLRLPGAWVHGGL  742
             LY    R I+ P L  + +  +C   A  KD   V        LLE +R    W   G 
Sbjct  483  TLYEYSFRQIVSPALSNLVQRLICEAEAEHKDVCRVVTCFSTDMLLEEMRTIQLW---GK  539

Query  743  DCMELALDQADGPEQISTMPAEILSPPPLIRHPNNISPSESLSFMPTSSTAKMYTIESPS  802
            D      D A+            L   P I                TS+TA         
Sbjct  540  D-----YDWAN------------LQTSPTI----------------TSTTATF-------  559

Query  803  STDSISPSAPSSHTTSPIRTGSSPPTSLSPPSCAPTIALITPDPPIVSSTLSSVPSPKPN  862
              DS+S            R+  SP ++L+ PS +P  AL +P  P  +   ++  +    
Sbjct  560  --DSVSE-----------RSRGSPASTLTTPSPSPP-ALTSPKSP--AGVFAAAQNDTRP  603

Query  863  PAQNMKKIDPRFPLSPKTSRPVPQIPRVPLDASRLGLYTKDIIKRAWWDACDDMRRCVCS  922
            P  N+  +       P   R +  IP +P  +  +G  T+ +++ AW DA   + +C CS
Sbjct  604  PPPNVVVVAAAEAAMPLPDRYLDHIPHIPRASEHMGTNTRQLLEGAWRDAIAGLFKCQCS  663

Query  923  ICLRG  927
            ICLR 
Sbjct  664  ICLRA  668


>gb|ELU41967.1| hypothetical protein AG1IA_04003 [Rhizoctonia solani AG-1 IA]
Length=743

 Score = 63.2 bits (152),  Expect = 1e-06, Method: Compositional matrix adjust.
 Identities = 75/300 (25%), Positives = 125/300 (42%), Gaps = 79/300 (26%)

Query  702  LGQIARLCALQK---DPLNVAPKLGIHKLLELLRLPGAWVHGGLDCMELALDQADGP---  755
            L  I   C L +   DP  V  ++G+ +L+E+LR P  WV GG    ++ +D  D     
Sbjct  358  LESIRSQCVLSRGESDPCKVVSRIGVDRLVEILRTPEPWVEGG-GWEDVYVDWEDANSFR  416

Query  756  EQISTMPAEI--------------LSP----------------PPLIRHPNNISPS--ES  783
            ++ S  P+                ++P                P L   P++++P+  ES
Sbjct  417  DRDSATPSNFRESIAHGNFRDHGSVTPNNFRDLESANIPREREPSLA--PSSVAPTLRES  474

Query  784  LSFMPTSSTAK-------MYTIESPSSTDSISPSAPSSHTTSPIRTGSSPPTSLSPPSCA  836
            ++  P  ST         M +++SP  + +IS  +P+   +SP+ T  SP  +L+ PS  
Sbjct  475  VAPSPPPSTISAQSPPPTMASVQSPRPS-TISAPSPTDQRSSPVSTTPSP-EALTSPSTM  532

Query  837  PTIALITPDPP-------IVSSTLSSV------------------PSPKPNPAQNMKKID  871
             T    TP PP       ++S + + V                  P  + +         
Sbjct  533  RT----TPSPPPIIDGKRLISESDNYVTHSHADGFAHSQTDNFHHPHSRSHTRTGSFTTS  588

Query  872  PRFPLSPKTSRPVPQIPRVPLDASRLGLYTKDIIKRAWWDACDDMRRCVCSICLRGLKAQ  931
            PR P SP   +P+  IP VP+    LG  TK +++  W +A   +  C C+IC R ++ Q
Sbjct  589  PRLPTSPILKKPLHPIPLVPISGYYLGQQTKVVLEMLWREATGGLWMCRCTICTRAMQVQ  648


>gb|KII95436.1| hypothetical protein PLICRDRAFT_34291 [Plicaturopsis crispa FD-325 
SS-3]
Length=752

 Score = 61.2 bits (147),  Expect = 5e-06, Method: Compositional matrix adjust.
 Identities = 32/88 (36%), Positives = 46/88 (52%), Gaps = 2/88 (2%)

Query  843  TPDPPIVSSTLSSVPSPKPNPAQNMKKIDPRFPLSPKTSRP--VPQIPRVPLDASRLGLY  900
            T  P + +STL + PSP P   +    I    P+SP    P  + QIP VP+ A+ L  Y
Sbjct  435  TTSPVLSTSTLQTTPSPSPTAEKGEGPITIPIPVSPVLEAPQLLHQIPYVPVTAAHLPQY  494

Query  901  TKDIIKRAWWDACDDMRRCVCSICLRGL  928
            + D +K  W +AC  +  C CS+C R +
Sbjct  495  SSDALKTVWREACSPLYHCRCSVCERAM  522


 Score = 50.1 bits (118),  Expect = 0.015, Method: Compositional matrix adjust.
 Identities = 39/153 (25%), Positives = 64/153 (42%), Gaps = 16/153 (10%)

Query  604  IVEMATEAWIHQRVIDGFWVSPLEQRAFQETFSQQTPSSAEGDITLDVVHPLSSSTLGSS  663
            + E+A   W  QR++DGFW SP +Q      +    P     ++      P +S    + 
Sbjct  253  VRELALGEWARQRILDGFWFSPADQ-----WYRNVVPGHPPQEVRAVHPCPWNSEVDAAD  307

Query  664  VCTTSSHPN-----GPLPPAGGALDQKTINLYSRKLRSILLPGLGQIARLCALQ-----K  713
                  HP      GP PP+    +Q  I  + R +RSILL  +  + R   ++      
Sbjct  308  EENNVHHPREETLMGPPPPSFHLCEQAYI-AFQRAMRSILLAPMNNLVRKIVIECGADGV  366

Query  714  DPLNVAPKLGIHKLLELLRLPGAWVHGGLDCME  746
            DP   A ++ +  +L  LR      + G+D +E
Sbjct  367  DPGVRASRMDMEDVLRALREEEGLWYNGIDWLE  399


>gb|KIM86033.1| hypothetical protein PILCRDRAFT_86708 [Piloderma croceum F 1598]
Length=1022

 Score = 60.5 bits (145),  Expect = 9e-06, Method: Compositional matrix adjust.
 Identities = 82/345 (24%), Positives = 136/345 (39%), Gaps = 79/345 (23%)

Query  604  IVEMATEAWIHQRVIDGFWVSPLEQ------RAFQETFSQQTPSSAEGDITLDVVHPLSS  657
            + ++A   W   R++DG+W SP +Q      R      S   P+    D T +    +  
Sbjct  499  VKDLALNDWARNRLLDGYWTSPADQWYGYTVRGLPTAVSAIHPTPWNRDATYE--GAMED  556

Query  658  STLGSSVCTTSSHPN-----GPLPPAGGALDQKTINLYSRKLRSILLPGLGQIARLCALQ  712
            S  G  +     HP        +PP+    +Q   N + +++R ILLP +  I R   ++
Sbjct  557  SQGGEIL----DHPRPSTVEAEVPPSYHLCEQ-AFNAFQKQMRLILLPAMSNIVRRLVIE  611

Query  713  -----KDPLNVAPKLGIHKLLELLRLPGAWVHGGLDCMELALDQADGPEQISTMPAEILS  767
                  DP   A K+ + ++   LR   +W + G+D +E    +A+G  +  T  A+   
Sbjct  612  CGADGVDPAMRAIKMDMEEVARELRDEASW-YNGIDWLE---RRANGERERRTRIAK---  664

Query  768  PPPLIRHPNNISPSESLSFMPTSSTAKMYTIESPSSTDSISPSAPSSHTTSPIRTGSSPP  827
                          ES S   +S +     + S +ST   +PS P+S     I+  +  P
Sbjct  665  -----------EDDESSSSSKSSGSHSTSPVLS-TSTLGTTPSPPASVDGHDIKKDAELP  712

Query  828  TSLSPPSCAPTIALITPDPPIVSSTLSSVPSPKPNPAQNMKKIDPRFPLSPKTSRPV--P  885
             S S  + AP++ +                                 P+SP    PV   
Sbjct  713  ASRS--TRAPSVVI---------------------------------PISPVLESPVLLH  737

Query  886  QIPRVPLDASRLGLYTKDIIKRAWWDACDDMRRCVCSICLRGLKA  930
             IP +P+  SR+  Y+++  K  W +AC  +  C CSIC R   A
Sbjct  738  PIPYIPVTLSRMPQYSQEAFKLVWREACAPLYHCRCSICERATMA  782


>gb|KIR84382.1| hypothetical protein I308_05397 [Cryptococcus gattii IND107]
Length=1101

 Score = 60.5 bits (145),  Expect = 1e-05, Method: Compositional matrix adjust.
 Identities = 78/330 (24%), Positives = 125/330 (38%), Gaps = 53/330 (16%)

Query  205  AGGRNGKQWKASDLPLKIIRQIIHLVAISIVTEEVQAKLADGDNRIHFGKRPVGLTIAHI  264
            A G++   W   DLP++I+ +I+   A   V  +   +   G N     +R   + +A +
Sbjct  54   AAGKDKAGW--GDLPVEILHRILSY-AQDAVPLDFTLQTYYGHN-----QRTREIALALV  105

Query  265  RARSRIAPLRLVCRAWKQATETIPFWSELATVF-----------------GFP---HNIR  304
            R R     +R+VC  W+ A ++ PFW E   +                   FP   H  R
Sbjct  106  R-RIWFCRMRMVCTGWRNAVDSHPFWPEFTLLLDPSRHHASTISDIPPPASFPTLFHRAR  164

Query  305  PYRENDCLITQLCWVCHFARFPLSQPAAYVRTTLGTVWICKLHLAVPFCGRCLRSRIDPA  364
                + CL  +L        +P  +      +       C+ H A  FC  C+R     A
Sbjct  165  SSTLSSCLACRLNRPSRLGHYPAVRKRLTFTSKFALAPTCEKH-ANSFCSGCMRESEPQA  223

Query  365  FRS-----RRLQWIGPRSSLPEYTAPNEDIDPGLRLTEDDDVEDEDDDINGGVNAGRELY  419
             R+     R L  IG R    E  +P+E   P L L++       D D NG     R L 
Sbjct  224  DRAPSPVLRHLTDIGARVESGETRSPSEPAVPPLMLSQ---CNHGDVDENGVERFPRSLV  280

Query  420  GPN---MACCQRCR---YECLQEAVTKRDPAVRIDLEDNGDLECFFQYVERAQGSAESIA  473
             P+    A     R   +EC +    + + ++ +  E   D      Y+    G+A  + 
Sbjct  281  CPDCRKAAIWNEIRLILHECARGGRMRGERSLWLYNEKVKD------YINFNVGTAFEMG  334

Query  474  VDLLERRWL---ARWTKFHDLATQVVQTEK  500
               +E +WL    RW +  + A Q+   EK
Sbjct  335  YAAVEEQWLIDHTRWVELSETALQLQNHEK  364


>gb|KIJ63498.1| hypothetical protein HYDPIDRAFT_29304 [Hydnomerulius pinastri 
MD-312]
Length=1084

 Score = 59.3 bits (142),  Expect = 2e-05, Method: Compositional matrix adjust.
 Identities = 158/732 (22%), Positives = 244/732 (33%), Gaps = 179/732 (24%)

Query  300  PHNIRPYRENDCLITQLCWVCHF-ARFPLSQPAAYVRT----TLGTVWICKLHLAVPFCG  354
            P+ + P+R    + T  C VC   + F     AA  R      +G + +C+ H    FCG
Sbjct  157  PYRLTPFRHFRQMATHSCVVCRINSPFSSVGLAAAKRPHHVPVIGAITLCREHRKTTFCG  216

Query  355  RCLR----SRIDPAF----RSRRL----QWIGPRSSLP----EYTAPNEDIDPGLRLTED  398
             CLR    +  DP F     + R     Q  GP + L      Y      ++P L +   
Sbjct  217  VCLREAPPAEFDPQFDIQGNTMRFSGYNQPYGPAAHLSIGMGGYNPYPTALNPALMVC--  274

Query  399  DDVEDEDDDINGGVNAGRELYGPNMACCQRCRYECL------------------------  434
               E+EDD+   GV+            C+ CR E L                        
Sbjct  275  -CAENEDDETWPGVDM----------TCRSCRSEGLWQRISTSLRDREAVGGPRFVPSTS  323

Query  435  ----------QEAVTKRDPAVRIDLEDNGDLECFFQYVERAQGSAESIAVDLLERRWLAR  484
                      Q     R P  +    D         +++  +GS   +     E+ WL  
Sbjct  324  QGSSRSIKDRQGTTISRHPPAKYASADWETRSTVDAFIDLGEGSITEVINVAREKFWLRS  383

Query  485  WTKFHDLATQVVQTEKINPVTAASADGNDMR-----AIFKASMGIEGDGSEPMTEALERE  539
             TK  +L  Q V   +     A    G D          +AS  +E  G     E     
Sbjct  384  QTKMPELMEQAVAATRW----AGEGMGVDFTPHTPAVNNQASTHLESSGGAAAPETAVVN  439

Query  540  LAQ---FEYEAAVAREENAARVVGAEHNNSDGERQTDEEGQGQEDEEEEEE----EEEEE  592
            +A      Y  A +   +      A+ + S   R          +EEE+ E     E+  
Sbjct  440  VANDVAESYSPANSTSPSPPSPRSAQRSRSPSPRSVYSSEYEGSEEEEDTELLSLTEDAG  499

Query  593  GVELTPGGYIQIVEMATEAWIHQRVIDGFWVSPLEQR-------------AFQETFSQQT  639
            GV           E+A   W   R++DG W SP +Q              A +ET S+  
Sbjct  500  GVR----------ELAISDWTRSRILDGHWCSPADQWYGHAWPAGGAVRDAGEETESEPD  549

Query  640  P-------SSAEGDITLDVVHPLS-SSTLGSSVCTTSSHPNGPLP---------PAGGAL  682
            P       SS      +  VHP   + ++ SS  + +S  +   P         P    L
Sbjct  550  PDEEMREGSSVRRTTRVPAVHPCPWTISIPSSPASPTSEADNVHPRMSTVRAHAPPSFTL  609

Query  683  DQKTINLYSRKLRSILLPGLGQIAR------LCALQKDPLNVAPKLGIHKLLELLRLPGA  736
             ++    Y ++L+ IL+P +  I R         +  DP     ++ + ++L  LR PG 
Sbjct  610  CEQAYRAYQKQLQLILIPAMTNIVRRFVIECTSGIAGDPAVRVARMNLEEVLAELRTPGV  669

Query  737  WVHGGLDCMELALDQADGPEQISTMPAEILSPPPLIRHPNNISPSESLSFMPTSSTAKMY  796
            W  G        +D A G    +    +            +     S +  P  ST  + 
Sbjct  670  WFDG--------VDWASGGLGGNRGREQEREREEDSASSASSRSDGSHTTSPVLSTTTLQ  721

Query  797  TIESPSSTDSISPSAPSSHTTSPIRTGSSPPTSLSPPSCAPTIALITPDPPIVSSTLSSV  856
            T          +PS P +H     +   S  TS++     P+  +  P  P++       
Sbjct  722  T----------TPSPPPAHAEDKGKEKHSEGTSVT--RQLPSAVIPIPVSPVL-------  762

Query  857  PSPKPNPAQNMKKIDPRFPLSPKTSRPVPQIPRVPLDASRLGLYTKDIIKRAWWDACDDM  916
                    Q+ K + P  P  P T   V  + R  L+A           K AW +AC  +
Sbjct  763  --------QSPKLLHP-IPYIPVT---VAHMARYSLEA----------FKMAWREACAPL  800

Query  917  RRCVCSICLRGL  928
              C CSIC R +
Sbjct  801  FHCRCSICERAM  812


>gb|KDQ28459.1| hypothetical protein PLEOSDRAFT_173647 [Pleurotus ostreatus PC15]
Length=828

 Score = 58.9 bits (141),  Expect = 2e-05, Method: Compositional matrix adjust.
 Identities = 90/386 (23%), Positives = 145/386 (38%), Gaps = 87/386 (23%)

Query  555  AARVVGAEHNNSDGERQTDEEGQGQEDEEEEEEEEEEEGVELTPGGYIQIVEMATEAWIH  614
            A+R VG E+  +D +    E    +E++ +  +  EE G          + E+A   W  
Sbjct  291  ASRYVGYENEAADDDLTLSELEAEEEEDADLMQMTEESG---------GVRELAISDWAR  341

Query  615  QRVIDGFWVSPLEQRAFQETFSQQTPSSAEGDITLDVVHPLSSSTLGSSVCTTSS--HP-  671
             R++DGFW SP +Q  +   F    P        +  VHP   +   S V    +  HP 
Sbjct  342  NRILDGFWWSPADQ--YYGNFVPNRPR------VVRAVHPCPWALDESEVQRDGAEDHPR  393

Query  672  ----NGPLPPAGGALDQKTINLYSRKLRSILLPGLGQIARLCALQ-----KDPLNVAPKL  722
                   +PP+  AL ++  N + R+ R+IL P +  I R   ++      DP   A ++
Sbjct  394  QAIVTADIPPS-YALCEQAYNAFQRQQRTILFPAMRNIVRKLVIECGADGVDPAVKAARM  452

Query  723  GIHKLLELLRLPGAWVHGGLDCMELALDQADGPEQISTMPAEILSPPPLIRHPNNISPSE  782
                +L  LR    W + G+D +E   +     E+                         
Sbjct  453  TPEDVLAELRDEAVW-YDGIDWLEKRRNARRDQEE-------------------------  486

Query  783  SLSFMPTSSTAKMYTI-ESPSSTDSISPSAPSSHTTSPIRTGSSPPTSLSPPSCAPTIAL  841
                      AK+  + E  S   S S  +  SHTTSP+ + S+  T             
Sbjct  487  ----------AKLRRLREEGSDDSSSSTKSDGSHTTSPVLSTSTLQT-------------  523

Query  842  ITPDPPIVSSTLSSVPSPKPNPAQNMKKIDPRFPLSPKTSRPVPQIPRVPLDASRLGLYT  901
             TP PP V +   S      N   ++ ++           R +  IP +P+  S L  Y+
Sbjct  524  -TPSPPPVDNKDDS------NIDTHLGRVTIAVAPVLDPPRLLRSIPYIPITISNLPQYS  576

Query  902  KDIIKRAWWDACDDMRRCVCSICLRG  927
             +  K  W +AC  +  C C +C R 
Sbjct  577  LEAFKAVWREACAPLYHCRCRVCERA  602


>ref|XP_001829909.2| hypothetical protein CC1G_04598 [Coprinopsis cinerea okayama7#130]
 gb|EAU91831.2| hypothetical protein CC1G_04598 [Coprinopsis cinerea okayama7#130]
Length=1229

 Score = 56.6 bits (135),  Expect = 1e-04, Method: Compositional matrix adjust.
 Identities = 166/788 (21%), Positives = 278/788 (35%), Gaps = 217/788 (28%)

Query  300  PHNIRPYRENDCLITQLCWVCHFARFPLSQPAAYV------RTT-----LGTVWICKLH-  347
            P  I  ++    ++   C+VC     P +     V      RTT     LG V +C+ H 
Sbjct  162  PVRISFWKHLRSILAVSCYVCRINAPPSNIGLGSVHHSKDRRTTMFTPYLGWVNVCRDHE  221

Query  348  -LAVPFCGRCLRSRIDPAFRSRRLQWIGPRSSLPEYTAPNEDIDPGLRLTEDDDVEDEDD  406
                 FCG CLR    P F S     +  +  L + +  +      L ++    +E+ED+
Sbjct  222  RRKAGFCGLCLREA--PIFESATANAVLSQYGLNQESQAHAQALGSLVMSVAC-IENEDE  278

Query  407  DINGGVNAGRELYGPNM-ACCQRCRYECL----------QEAV---------TKRD----  442
            +             PN+ A C+ CR E L          +EAV         +K D    
Sbjct  279  ET-----------WPNVEATCRTCRLEWLWRKTNTNERDREAVAGGAAPRTASKDDFPPN  327

Query  443  -PAVRI---DLEDNGDLECFFQYVERAQGSAESIAVDLLERRWLARWTKFHDLATQVVQT  498
              A+ +   D E    +ECF    E   G+   +     E+ WL ++TK  D+  Q +  
Sbjct  328  LAAILLSSEDWETRQTIECFLDLGE---GTINDVLCLAREKWWLRKFTKLGDMMQQALAA  384

Query  499  EKINPVTAASADGNDMRAIFKASMGIEGDGSEPMTEALERELAQFEYEAAVAREENAARV  558
             + N       +  DM      + G +    +     L  E A        +   + A  
Sbjct  385  RRWN------GNTEDMSGRASPARGYQDHQHQSQQVVLLEEAATITSRTLSSSGSDNASE  438

Query  559  VGAEHNNSDGERQTDEEGQGQEDEEEEEEEEEEEGVELTP-----------GGYIQ----  603
            +  E  + D    T +  Q +    E  ++ E  G E                 +Q    
Sbjct  439  MDREETHYDSADATPDH-QRRPILREVTDDYESLGDEEESLQDDDEDDDEDAALLQREEN  497

Query  604  -IVEMATEAWIHQRVIDGFWVSPLEQRAFQET--------FSQQTPSSAE---GDITLDV  651
             + E+A   W   R++DG+W+SP +     +T        + ++     E   G+     
Sbjct  498  SVKELALGDWARARILDGYWISPADTWYNLDTGYPPPSVRYQKEKQGGGEARKGETWTYA  557

Query  652  VHP----------------LSSSTLGSSVC-------------------TTSSHP-----  671
            VHP                + ++  GSS                        SHP     
Sbjct  558  VHPCPWTVEKESDGDSSRVVDTAEAGSSSSDDHGESWENQEDDKRMDEEQEDSHPRWSVV  617

Query  672  NGPLPPAGGALDQKTINLYSRKLRSILLPGLGQIARLCALQ-------------------  712
               +PP+    +Q  +  + ++LR ILLP +  I R   ++                   
Sbjct  618  TAEVPPSYTLCEQAFL-AHQKQLRLILLPAMRNIVRKLVMECGAAATSSDLAYQVTHTKK  676

Query  713  ---KDPLNVAPKLGIHKLLELLRLP-GAWVHGGLDCMELALDQ-----------------  751
               +DP  +A ++ + +++ +LR   G W   G+D +E   ++                 
Sbjct  677  RKLEDPAIIASRMSMEEVVRILREEEGVWF-DGVDWIERMKNREADEGAGVRKRPRASYD  735

Query  752  ---ADGPEQISTMPAEILSPPPLIRHPNNISPSESLSFMPTSSTAKMYTIESPSSTDSIS  808
               ADG    +  P E+++ P    H +  +  +         TAK  +  S S +    
Sbjct  736  AHDADG---TANEPEEVVNSP----HSSPTTDDDDRDPYRPGHTAKAASASSGSGS----  784

Query  809  PSAPSSHTTSPIRTGSSPPTSLSPPSCAPTIAL----------ITPDPPIVSSTLSSVPS  858
                    TSP+ + ++  T+ SPP  AP++AL          ++ D  I     S    
Sbjct  785  -------GTSPVLSTTTLETTPSPP-MAPSMALSSEDASNKAAVSRDESIEDGDASEDGE  836

Query  859  PKPNPAQNMKKIDPRFPL--SPKTSRPVPQIPRVPLDASRLGLYTKDIIKRAWWDACDDM  916
            P P      K + P  P+  +P+  RP   IP VP   +R   YT + +K  W +AC  +
Sbjct  837  PVP------KILIPVAPVLEAPRLLRP---IPYVPTTVARFPQYTMEALKMVWREACVPL  887

Query  917  RRCVCSIC  924
              C CSIC
Sbjct  888  FHCRCSIC  895


>gb|EMD32918.1| hypothetical protein CERSUDRAFT_118352 [Ceriporiopsis subvermispora 
B]
Length=813

 Score = 56.2 bits (134),  Expect = 2e-04, Method: Compositional matrix adjust.
 Identities = 83/348 (24%), Positives = 131/348 (38%), Gaps = 81/348 (23%)

Query  604  IVEMATEAWIHQRVIDGFWVSPLEQ-RAFQETFSQQTPSSAEGDITLDVVHPL---SSST  659
            + E+A + W   R++DG+WVSP +Q   F  T     PS          VHP      +T
Sbjct  328  VKELAIQDWARNRILDGYWVSPADQWYGFANTSRPLVPS----------VHPCPWNRGAT  377

Query  660  LGSSVCTTSS--------HPN----GPLPPAGGALDQKTINLYSRKLRSILLPGLGQIAR  707
               +V    +        HP     G  PP    L + T   Y +++  ILLP +  I R
Sbjct  378  YSGAVDDGETEGDGAELDHPRPRTVGAPPPPTYQLCEHTYRAYIKQMHDILLPAMRNIVR  437

Query  708  LCALQ-----KDPLNVAPKLGIHKLLELLRLPGAWVHGGLDCMELALDQADGPEQISTMP  762
              A++      DP   A ++   ++L  L     W + G+D +E   ++           
Sbjct  438  RIAIECSADGADPTIRASRMTTEEVLRELHHESTWFN-GIDWLERRANE-----------  485

Query  763  AEILSPPPLIRHPNNISPSESLSFMPTSSTAKMYTIESPSSTDSI--SPSAPSSHTTSPI  820
             E+       R        +      +S +   +T     ST ++  +PS P S      
Sbjct  486  RELERKRQRERKRGKGKEKDEDESSSSSRSEGSHTTSPVLSTTTLQTTPSPPPSGEAKED  545

Query  821  RTGSSPPTSLSPPSCAPTIALITPDPPIVSSTLSSVPSPKPNPAQNMKKIDPRFPLSPKT  880
             T  SP TS++P                   TLS   SP       +K        SP+ 
Sbjct  546  ETVVSPTTSVAP-------------------TLSIAVSPV------LK--------SPEL  572

Query  881  SRPVPQIPRVPLDASRLGLYTKDIIKRAWWDACDDMRRCVCSICLRGL  928
              P+P I   P+  S++  Y  D   + W +AC  + +C CS+C R +
Sbjct  573  IHPIPYI---PVALSQMPQYGLDAFTQVWREACAPLYQCRCSVCERAV  617


>gb|KEP52424.1| hypothetical protein V565_045870 [Rhizoctonia solani 123E]
Length=844

 Score = 55.5 bits (132),  Expect = 3e-04, Method: Compositional matrix adjust.
 Identities = 70/251 (28%), Positives = 108/251 (43%), Gaps = 49/251 (20%)

Query  709  CAL---QKDPLNVAPKLGIHKLLELLRLPGAWVHGGLDCMELALDQADGPEQISTMPAEI  765
            C L   + DP     ++   +L+ELL+ P  WV GG    ++ +D     E  STM  E 
Sbjct  526  CVLAHGEPDPCKAVSRINADRLVELLKGPEPWVEGG-GWEDIYVDWE---EPDSTMVFER  581

Query  766  LSPPPLIRHPN----NISPS--ESLSFMPTSSTAKMYTIESPSSTDSISPSAPSSHTTSP  819
                P  R P+    +++P+  ES++  P  ST    +  SP  +D    S      +SP
Sbjct  582  DHDQPREREPSLTPSSVAPTLRESVAPSPPPST---ISAASPHPSDLQQSSPSDLQRSSP  638

Query  820  IRTGSSPPTSLSPPSCAPTIALITPDPPIVS-------------------STLSSVPSPK  860
            + T  SP  +L+ PS   T    TP PP +                    +T   VP+  
Sbjct  639  VSTTPSP-EALTSPSTMRT----TPSPPPMDGKKEEGDGVFLTRTTEGGFATRGFVPTAS  693

Query  861  PNPAQNMKKIDPRFPLSPKTSRPVPQIPRVPLDASRLGLYTKDIIKRAWWDACDDMRRCV  920
            P     ++ I PR      TS+P+  IP +P     LG +TK +++  W +A   +  C 
Sbjct  694  P-----VQPISPRL----STSKPLHPIPLIPHCTYHLGQHTKSVLEMLWREATGGLWMCR  744

Query  921  CSICLRGLKAQ  931
            C+IC R +  Q
Sbjct  745  CTICSRAMSVQ  755


 Score = 45.1 bits (105),  Expect = 0.42, Method: Compositional matrix adjust.
 Identities = 80/363 (22%), Positives = 121/363 (33%), Gaps = 100/363 (28%)

Query  205  AGGRNGKQWKASDLPLKIIRQIIHLVAISIVTEEVQAKLADGDNRIHFGKRPVGLTIAHI  264
            +G R GK+  ++ LP   IR II    +++    +   L D   ++      +GL +  +
Sbjct  9    SGMRKGKR-TSAVLPRVAIRLIIDWT-VTVDRAYIPRSLGDWSQQVW-----IGLHVGRV  61

Query  265  RARSRIAPLRLVCRAWKQATETIPFWSELATVF--------------------GFPHNIR  304
                 +  LR V R W++  +  P W  L  +                       P N  
Sbjct  62   -----LNTLRRVSRGWREVADEHPTWRTLCMILDPYLVLARPRVPLNPTQVPSQPPLNAD  116

Query  305  PYRENDCLITQLCWVCH------------FARFPLSQPAAYVRTTLGTVWICKLHLAVPF  352
             +R    +I   C VC              AR P S       +  G +  C +H  + F
Sbjct  117  AFRR---IIRATCLVCAVSCPDNRVYRHLMARGPQSLTYPVPSSMWGIIPACTVHRPLSF  173

Query  353  CGRCLRSRIDPAFRSRRLQWIGPRSSLPEYTAPNEDIDPGLRLTEDDDVEDEDDDINGGV  412
            CGRCLRS  +PA              L  YTAP+  + P L   E D       D+    
Sbjct  174  CGRCLRS--EPA-----------SLGLAHYTAPHLALAP-LAANERDPEFVRCLDLRTSS  219

Query  413  NAGRELYGPNMACCQRCRYECLQEAVTK------------------RDPAVRIDLEDNGD  454
             AG        A C  CR E ++  + +                   D A    +  +G 
Sbjct  220  GAGVR------AVCGACRDERIRTELVRGRGANMAPWMGSGFWDWDYDGADVWSVPWDGG  273

Query  455  LE---------------CFFQYVERAQGSAESIAVDLLERRWLARWTKFHDLATQVVQTE  499
            L                   QYVE    +      ++ ER W+  WTK  ++   V  T 
Sbjct  274  LSRRDADRLLRSEEVEGVVTQYVEYGDWTVREAIEEMEERVWMRWWTKASEMEGLVRATA  333

Query  500  KIN  502
            K+ 
Sbjct  334  KLQ  336


>gb|EUC57531.1| hypothetical protein RSOL_224210 [Rhizoctonia solani AG-3 Rhs1AP]
Length=845

 Score = 55.5 bits (132),  Expect = 3e-04, Method: Compositional matrix adjust.
 Identities = 70/251 (28%), Positives = 108/251 (43%), Gaps = 49/251 (20%)

Query  709  CAL---QKDPLNVAPKLGIHKLLELLRLPGAWVHGGLDCMELALDQADGPEQISTMPAEI  765
            C L   + DP     ++   +L+ELL+ P  WV GG    ++ +D     E  STM  E 
Sbjct  526  CVLAHGEPDPCKAVSRINADRLVELLKGPEPWVEGG-GWEDIYVDWE---EPDSTMVFER  581

Query  766  LSPPPLIRHPN----NISPS--ESLSFMPTSSTAKMYTIESPSSTDSISPSAPSSHTTSP  819
                P  R P+    +++P+  ES++  P  ST    +  SP  +D    S      +SP
Sbjct  582  DHDQPREREPSLTPSSVAPTLRESVAPSPPPST---ISAASPHPSDLQQSSPSDLQRSSP  638

Query  820  IRTGSSPPTSLSPPSCAPTIALITPDPPIVS-------------------STLSSVPSPK  860
            + T  SP  +L+ PS   T    TP PP +                    +T   VP+  
Sbjct  639  VSTTPSP-EALTSPSTMRT----TPSPPPMDGKKEEGDGVFLTRTTEGGFATRGFVPTAS  693

Query  861  PNPAQNMKKIDPRFPLSPKTSRPVPQIPRVPLDASRLGLYTKDIIKRAWWDACDDMRRCV  920
            P     ++ I PR      TS+P+  IP +P     LG +TK +++  W +A   +  C 
Sbjct  694  P-----VQPISPRL----STSKPLHPIPLIPHCTYHLGQHTKSVLEMLWREATGGLWMCR  744

Query  921  CSICLRGLKAQ  931
            C+IC R +  Q
Sbjct  745  CTICSRAMSVQ  755


 Score = 45.1 bits (105),  Expect = 0.45, Method: Compositional matrix adjust.
 Identities = 80/363 (22%), Positives = 121/363 (33%), Gaps = 100/363 (28%)

Query  205  AGGRNGKQWKASDLPLKIIRQIIHLVAISIVTEEVQAKLADGDNRIHFGKRPVGLTIAHI  264
            +G R GK+  ++ LP   IR II    +++    +   L D   ++      +GL +  +
Sbjct  9    SGMRKGKR-TSAVLPRVAIRLIIDWT-VTVDRAYIPRSLGDWSQQVW-----IGLHVGRV  61

Query  265  RARSRIAPLRLVCRAWKQATETIPFWSELATVF--------------------GFPHNIR  304
                 +  LR V R W++  +  P W  L  +                       P N  
Sbjct  62   -----LNTLRRVSRGWREVADEHPTWRTLCMILDPYLVLARPRVPLNPTQVPSQPPLNAD  116

Query  305  PYRENDCLITQLCWVCH------------FARFPLSQPAAYVRTTLGTVWICKLHLAVPF  352
             +R    +I   C VC              AR P S       +  G +  C +H  + F
Sbjct  117  AFRR---IIRATCLVCAVSCPDNRVYRHLMARGPQSLTYPVPSSMWGIIPACTVHRPLSF  173

Query  353  CGRCLRSRIDPAFRSRRLQWIGPRSSLPEYTAPNEDIDPGLRLTEDDDVEDEDDDINGGV  412
            CGRCLRS  +PA              L  YTAP+  + P L   E D       D+    
Sbjct  174  CGRCLRS--EPA-----------SLGLAHYTAPHLALAP-LAANERDPEFVRCLDLRTSS  219

Query  413  NAGRELYGPNMACCQRCRYECLQEAVTK------------------RDPAVRIDLEDNGD  454
             AG        A C  CR E ++  + +                   D A    +  +G 
Sbjct  220  GAGVR------AVCGACRDERIRTELVRGRGANMAPWMGSGFWDWDYDGADVWSVPWDGG  273

Query  455  LE---------------CFFQYVERAQGSAESIAVDLLERRWLARWTKFHDLATQVVQTE  499
            L                   QYVE    +      ++ ER W+  WTK  ++   V  T 
Sbjct  274  LSRRDADRLLRSEEVEGVVTQYVEYGDWTVREAIEEMEERVWMRWWTKASEMEGLVRATA  333

Query  500  KIN  502
            K+ 
Sbjct  334  KLQ  336


>gb|KLO05862.1| hypothetical protein SCHPADRAFT_910743 [Schizopora paradoxa]
Length=831

 Score = 53.9 bits (128),  Expect = 8e-04, Method: Compositional matrix adjust.
 Identities = 50/209 (24%), Positives = 84/209 (40%), Gaps = 30/209 (14%)

Query  548  AVAREENAARVVGAEHNNSDGERQTDEEGQGQEDEEEEEEEEEEEGVELTP-----GGYI  602
            AVA  +   R  G     ++G R+     +  E EEE  E ++E+  +L       GG  
Sbjct  274  AVATSKLQNRTTG----RNNGFRRLSMGAEQYESEEEMSEADDEDDPDLMSMTEDRGG--  327

Query  603  QIVEMATEAWIHQRVIDGFWVSPLEQRAFQETFSQQTPSSAEGDITLDVVHPLSSSTLGS  662
             + ++A   W   R+++G W+SP +Q         Q P        ++  HP   +   S
Sbjct  328  -VRDLAINDWARTRILEGHWLSPADQ---WYALQGQAPPEWGHPPYINATHPCPWNYPAS  383

Query  663  SVCTTSSHPNGPL------PPAGGALDQKTINLYSRKLRSILLPGLGQIARLCALQK---  713
                 +  P+ PL      PP    L       Y R++RS+L P +  I R   ++    
Sbjct  384  E----ADQPHPPLTMAFSSPPPTLPLCSSAFEAYKRQMRSLLYPAMVNIVRRIVMESAMD  439

Query  714  --DPLNVAPKLGIHKLLELLRLPGAWVHG  740
              D      K+ +  + + LR+ G W +G
Sbjct  440  GTDACVRVAKMEVEDIAKALRVSGVWYNG  468


>ref|XP_007316347.1| hypothetical protein SERLADRAFT_355167 [Serpula lacrymans var. 
lacrymans S7.9]
 gb|EGO26174.1| hypothetical protein SERLADRAFT_355167 [Serpula lacrymans var. 
lacrymans S7.9]
Length=997

 Score = 53.9 bits (128),  Expect = 0.001, Method: Compositional matrix adjust.
 Identities = 67/270 (25%), Positives = 91/270 (34%), Gaps = 78/270 (29%)

Query  273  LRLVCRAWKQATETIPFWSELATVFGFPHNI------------------------RPYRE  308
            L  V  AWK A ET  FW +  ++   PH+                          PYR 
Sbjct  104  LMRVSPAWKAALETHLFWYQACSIID-PHDTLAQHIHTNPLIPSHHSSHAPRQTPSPYRH  162

Query  309  NDCLITQLCWVCHFARFPLSQPAAYVRT------------TLGTVWICKLHLAVPFCGRC  356
               L +  C VC       + PA Y+ T            TLG V +CK H    FCG C
Sbjct  163  FRTLTSTSCIVCLIN----APPATYISTGLFLAKRTHPTPTLGVVTLCKDHRRAFFCGVC  218

Query  357  LRSRIDPAFRSRRLQWIGPRSSLPEYTAPNEDIDPGLRLTEDDDVEDEDDDINGGVNAGR  416
            LR                P S     T P   I P L +      E+EDD+   GV    
Sbjct  219  LRE--------------APVSECEPETGPYAPIHPSLMVC---CAENEDDETWPGVE---  258

Query  417  ELYGPNMACCQRCRYECLQEAV--TKRDPAV--------RIDLEDNGDLECFFQYVERAQ  466
                     C+ CR E L   +  ++RD                D    +    ++E  +
Sbjct  259  -------TTCRSCRTEFLWRRISGSERDREAIGGALWPPTFHSTDYQTRQTVDAFIELGE  311

Query  467  GSAESIAVDLLERRWLARWTKFHDLATQVV  496
            GS   +     E+ WL + TK  +L  Q +
Sbjct  312  GSISEVLCVAREKLWLRKETKMVELMGQAL  341


>ref|XP_007398245.1| hypothetical protein PHACADRAFT_259980 [Phanerochaete carnosa 
HHB-10118-sp]
 gb|EKM53558.1| hypothetical protein PHACADRAFT_259980 [Phanerochaete carnosa 
HHB-10118-sp]
Length=526

 Score = 53.1 bits (126),  Expect = 0.001, Method: Compositional matrix adjust.
 Identities = 53/183 (29%), Positives = 78/183 (43%), Gaps = 27/183 (15%)

Query  577  QGQEDEE-EEEEEEEEEGVELT--PGGYIQIVEMATEAWIHQRVIDGFWVSPLEQR----  629
            +G  DE+   EEEE+ E + LT   GG   I ++A   W   RV+DG WV+P +Q     
Sbjct  47   EGDSDEDLSGEEEEDPELMSLTEDAGG---IRDLAITDWARNRVLDGHWVAPADQWYDNS  103

Query  630  --AFQETFSQQTPSSAEGDITLDVVHPLSSSTLGSSVCTTSSHP-----NGPLPPAGGAL  682
                 +  S Q P    G      +    S+  G  +     HP     N   PP   AL
Sbjct  104  VPGQPDVVSAQHPCPWHGATYSGALEEGESAGDGEEL----EHPRPRTYNAACPPT-FAL  158

Query  683  DQKTINLYSRKLRSILLPGLGQIARLCALQK-----DPLNVAPKLGIHKLLELLRLPGAW  737
             +     Y R +R ILLP +  I R   ++      DP   A K+ + ++++ LR+   W
Sbjct  159  CEVVYRAYQRVMREILLPAMTNIVRRILMESQADGVDPTLRASKMTLEEVVKELRVHATW  218

Query  738  VHG  740
              G
Sbjct  219  SKG  221


 Score = 48.5 bits (114),  Expect = 0.030, Method: Compositional matrix adjust.
 Identities = 28/96 (29%), Positives = 48/96 (50%), Gaps = 11/96 (11%)

Query  846  PPIVSSTLSSVPSPKPNPAQN-------MKKIDPRFPLSP----KTSRPVPQIPRVPLDA  894
            P + ++TL + PSP P+  ++       +    P  P+S     +T   +  +P VP+  
Sbjct  261  PVLSTTTLQTTPSPPPSSVKDDDPESSPVAVSSPTIPVSEAAALQTHELIRPVPYVPVTV  320

Query  895  SRLGLYTKDIIKRAWWDACDDMRRCVCSICLRGLKA  930
            + L  Y+ D I+  W +AC  + +C CSIC R + A
Sbjct  321  AHLPQYSIDCIRTIWREACAPLYQCRCSICERAMLA  356


>ref|XP_006460529.1| hypothetical protein AGABI2DRAFT_220564, partial [Agaricus bisporus 
var. bisporus H97]
 gb|EKV48668.1| hypothetical protein AGABI2DRAFT_220564, partial [Agaricus bisporus 
var. bisporus H97]
Length=821

 Score = 53.1 bits (126),  Expect = 0.001, Method: Compositional matrix adjust.
 Identities = 64/280 (23%), Positives = 99/280 (35%), Gaps = 70/280 (25%)

Query  273  LRLVCRAWKQATETIPFWSELATV---------FGF----------------PHNIRPYR  307
            L  VC  W  A ET  FW +  T+         F                  P+ I PYR
Sbjct  82   LMAVCPEWDLALETHLFWQQAVTLVDPTDFLGQFAIITPHPDLAKVSPVPLRPYRITPYR  141

Query  308  ENDCLITQLCWVCHFARFPLSQP--AAYVRTT----LGTVWICK--LHLAVPFCGRCLRS  359
                +I   C VC    FP +    A   RT     LG V IC+   H  + FCG CLR 
Sbjct  142  HFRHIIGLSCLVCR-VNFPTTTRGLACAKRTAGTYILGYVPICRDHDHRRLAFCGLCLRE  200

Query  360  RIDPAFRSRRLQWIGPRSSLPEYTAPNEDIDPGLRLTEDDDVEDEDDDINGGVNAGRELY  419
                                           P   +   +     D  +    N  +E++
Sbjct  201  ------------------------------SPTHEMASANVYASNDLQVGVSKNDDKEMW  230

Query  420  GPNMACCQRCRYECL--QEAVTKRDP----AVRIDLEDNGDLECFFQYVERAQGSAESIA  473
                A C+ CR+E L  + ++  RD      + +  +D    +    +VE A+G+  ++ 
Sbjct  231  PGVDATCRNCRHEWLWKKASLNSRDQEAIGGIDLSSDDWETRQTVEGFVEMAEGTINAVI  290

Query  474  VDLLERRWLARWTKFHDLATQVVQTEKINPVTAASADGND  513
                E+ WL ++T+  D+  Q +   K N   + S+   D
Sbjct  291  NLAREKYWLRKFTRLDDMMQQALAAAKFNGYPSRSSSVRD  330


>emb|CDO72665.1| hypothetical protein BN946_scf184985.g84 [Trametes cinnabarina]
Length=889

 Score = 52.4 bits (124),  Expect = 0.003, Method: Compositional matrix adjust.
 Identities = 51/199 (26%), Positives = 86/199 (43%), Gaps = 52/199 (26%)

Query  574  EEGQGQEDEEE--EEEEEEEEGVELT--PGGYIQIVEMATEAWIHQRVIDGFWVSPLEQ-  628
            E G+G   +EE  +++ E+ E + LT   GG   I E+A   W   R++DG W+SP ++ 
Sbjct  333  ESGEGYNSDEELSDDDMEDPEMLSLTEDAGG---IRELAINDWARNRILDGHWISPADEW  389

Query  629  ----------------------RAFQETFSQQTPSSAEGDITLDVVHPLSSSTLGSSVCT  666
                                  RA  E   ++     +GD   ++ HP   + L      
Sbjct  390  FNNTKNGRPRLAPARHPCPWNRRAIYEGALEE----GQGDEAQELEHPRPKTHL------  439

Query  667  TSSHPNGPLPPAGGALDQKTINLYSRKLRSILLPGLGQIARLCALQ-----KDPLNVAPK  721
                   P PP+  AL ++T   ++R++R ILLP +  I R   ++      DP   A +
Sbjct  440  ------APHPPS-FALCEQTYRAFTRQMREILLPPMRNIVRKLVMECAADGTDPALKAVR  492

Query  722  LGIHKLLELLRLPGAWVHG  740
            + +  ++  LR   AW +G
Sbjct  493  MSVEDVMCELRDESAWYNG  511


 Score = 46.6 bits (109),  Expect = 0.16, Method: Compositional matrix adjust.
 Identities = 28/91 (31%), Positives = 45/91 (49%), Gaps = 8/91 (9%)

Query  846  PPIVSSTLSSVPSPKPNPAQN------MKKIDPRFPLSPKTSRP--VPQIPRVPLDASRL  897
            P + ++TL + PSP P+  ++           P  P+SP    P  +  IP VP+ A  +
Sbjct  552  PVLSTTTLHTTPSPPPSGKEDEAAASSPVAAAPPIPISPVLKAPELLRPIPYVPVTAEHM  611

Query  898  GLYTKDIIKRAWWDACDDMRRCVCSICLRGL  928
              Y+ +    AW +AC  + +C CSIC R +
Sbjct  612  PYYSVEAFVMAWREACTPLYQCQCSICERAM  642


>ref|XP_003197441.1| hypothetical protein CGB_N0490C [Cryptococcus gattii WM276]
 gb|ADV25654.1| Hypothetical Protein CGB_N0490C [Cryptococcus gattii WM276]
Length=1098

 Score = 52.4 bits (124),  Expect = 0.003, Method: Compositional matrix adjust.
 Identities = 68/329 (21%), Positives = 113/329 (34%), Gaps = 57/329 (17%)

Query  205  AGGRNGKQWKASDLPLKIIRQIIHLVAISIVTEEVQAKLADGDNRIHFGKRPVGLTIAHI  264
            A G++   W   DLP++I+ +I+     ++  +         D+R         + +A +
Sbjct  53   AAGKDKAGW--GDLPVEILHRILSFAQDAVPLDFTLQTYYGHDHRTR------AIALALV  104

Query  265  RARSRIAPLRLVCRAWKQATETIPFWSELATVF--------------------GFPHNIR  304
            R R     +R+VC  W+ A ++ PFW E   +                        H  R
Sbjct  105  R-RIWFCRMRMVCTGWRHAVDSHPFWPEFTLLLDPSRHHSSAISTTPPPPPFPTLFHRAR  163

Query  305  PYRENDCLITQLCWVCHFARFPLSQPAAYVRTTLGTVWICKLHLAVPFCGRCLRSRIDPA  364
                  CL  +L        +P  +      +       C+ H +  FC  C+R     A
Sbjct  164  SSTLASCLACRLNRPSRLGYYPAVRKRLTFTSKFALAPTCEKH-SNNFCSGCMRESEPQA  222

Query  365  FRSRRLQWIGPRSSLPEYTAPNEDIDPGLRLTEDDDVEDEDDDINGGVNAGRELYGPNMA  424
             R+     IG      E  +P+E   P L L++       D D NG     R L  P+  
Sbjct  223  GRAPSPG-IGALVEAGETRSPSEPAVPALMLSQ---CNQGDVDENGVERFPRSLVCPD--  276

Query  425  CCQRCRYECLQEAVTKRDPAVRIDLEDNGDL----------ECFFQYVERAQGSAESIAV  474
                    C + A+      +  D    G +          E    Y+    G+A  +  
Sbjct  277  --------CRKAAIWNEIRLILHDCARGGRMRGERSLWLYNEKVKDYINFNVGTAFEMGY  328

Query  475  DLLERRWL---ARWTKFHDLATQVVQTEK  500
              +E +WL    RW +  + A Q+   EK
Sbjct  329  AAVEEQWLIDHTRWVELSETALQLQNHEK  357


>gb|KIY32732.1| hypothetical protein I305_04890 [Cryptococcus gattii E566]
 gb|KJE01875.1| hypothetical protein I311_04514 [Cryptococcus gattii NT-10]
Length=1099

 Score = 52.4 bits (124),  Expect = 0.003, Method: Compositional matrix adjust.
 Identities = 68/329 (21%), Positives = 113/329 (34%), Gaps = 57/329 (17%)

Query  205  AGGRNGKQWKASDLPLKIIRQIIHLVAISIVTEEVQAKLADGDNRIHFGKRPVGLTIAHI  264
            A G++   W   DLP++I+ +I+     ++  +         D+R         + +A +
Sbjct  53   AAGKDKAGW--GDLPVEILHRILSFAQDAVPLDFTLQTYYGHDHRTR------AIALALV  104

Query  265  RARSRIAPLRLVCRAWKQATETIPFWSELATVF--------------------GFPHNIR  304
            R R     +R+VC  W+ A ++ PFW E   +                        H  R
Sbjct  105  R-RIWFCRMRMVCTGWRHAVDSHPFWPEFTLLLDPSRHHSSAISTTPPPPPFPTLFHRAR  163

Query  305  PYRENDCLITQLCWVCHFARFPLSQPAAYVRTTLGTVWICKLHLAVPFCGRCLRSRIDPA  364
                  CL  +L        +P  +      +       C+ H +  FC  C+R     A
Sbjct  164  SSTLASCLACRLNRPSRLGYYPAVRKRLTFTSKFALAPTCEKH-SNNFCSGCMRESEPQA  222

Query  365  FRSRRLQWIGPRSSLPEYTAPNEDIDPGLRLTEDDDVEDEDDDINGGVNAGRELYGPNMA  424
             R+     IG      E  +P+E   P L L++       D D NG     R L  P+  
Sbjct  223  GRAPSPG-IGALVEAGETRSPSEPAVPALMLSQ---CNQGDVDENGVERFPRSLVCPD--  276

Query  425  CCQRCRYECLQEAVTKRDPAVRIDLEDNGDL----------ECFFQYVERAQGSAESIAV  474
                    C + A+      +  D    G +          E    Y+    G+A  +  
Sbjct  277  --------CRKAAIWNEIRLILHDCARGGRMRGERSLWLYNEKVKDYINFNVGTAFEMGY  328

Query  475  DLLERRWL---ARWTKFHDLATQVVQTEK  500
              +E +WL    RW +  + A Q+   EK
Sbjct  329  AAVEEQWLIDHTRWVELSETALQLQNHEK  357


>gb|KIM36812.1| hypothetical protein M413DRAFT_284939 [Hebeloma cylindrosporum 
h7]
Length=663

 Score = 52.0 bits (123),  Expect = 0.003, Method: Compositional matrix adjust.
 Identities = 61/268 (23%), Positives = 103/268 (38%), Gaps = 73/268 (27%)

Query  273  LRLVCRAWKQATETIPFWSELAT--------VFGFPHNIRPYRENDC-------------  311
            L  +C++W++A E   FW+ +          +F    + +P  EN               
Sbjct  83   LMFICQSWRRAIEKHFFWNHIVNIIDPNDFLIFHMTVHPKPQHENASTIPSPIRLTYHQH  142

Query  312  ---LITQLCWVCHFARFPLSQPA------AYVRTTLGTVWICKLH--LAVPFCGRCLRSR  360
               +I   C VC    +P S         + + T LGT+ ICK H      +CG CLR  
Sbjct  143  LKNVIASSCVVCRI-NYPTSTLGLAAAKRSMITTYLGTLGICKDHDRRRASYCGLCLRDT  201

Query  361  IDPAFRSRRLQWIGPRSSLPEYTAPNEDIDPGLRLTEDDDVEDEDDDINGGVNAGRELYG  420
            +               +S P+ TA + ++   + +     VE+ED+D    V+       
Sbjct  202  VV--------------TSGPDTTARDYELSQAIAI-----VENEDEDTWPAVD-------  235

Query  421  PNMACCQRCRYECL----QEAVTKRDP--AVRIDLEDNGDLECFFQYVERAQGSAESIAV  474
               + C++CR E L      +V  R+      +  ED    +    ++E  +G+   +  
Sbjct  236  ---STCKKCRVEWLWRRANHSVRDREAIGGSSLQSEDWETRQTVENFIEMGEGTIGEVLA  292

Query  475  DLLERRWLARWTKF-----HDLATQVVQ  497
               E++WL   TK+     H LA QV  
Sbjct  293  LAREKQWLRLHTKYEALGQHMLAAQVTH  320


 Score = 43.1 bits (100),  Expect = 1.9, Method: Compositional matrix adjust.
 Identities = 38/165 (23%), Positives = 74/165 (45%), Gaps = 28/165 (17%)

Query  600  GYIQIVEMATEAWIHQRVIDGFWVSPLEQRAFQETFSQQTPSSAEGDITLDVVHPL----  655
            G  Q+ +++  AW  +R++DG W+SP +       ++   P      + +  +HPL    
Sbjct  349  GSGQVRDLSLTAWARERILDGHWISPADI-----YYAYTAPGQ---PLVVPTIHPLPWAR  400

Query  656  ----SSSTLGSSVCTTSSHPNGPLPPAGGALDQKTINLYSRKLRSILLPGLGQIARLCAL  711
                 S + G     T +     +PP     +Q  +  + + +R IL P +  I R   +
Sbjct  401  EVDPQSESNGEEQHPTRTTIAAEVPPTYTLCEQAYLA-HMKAMREILCPPMRNIVRKLVM  459

Query  712  Q---------KDPLNVAPKLGIHKLLELLR-LPGAWVHGGLDCME  746
            +         +DP ++A K+ +  +L++LR   G W + G+D +E
Sbjct  460  ECSVPTERGFEDPAHLARKMTLDDVLKVLREEEGVW-YDGVDWVE  503


>gb|KNZ74333.1| hypothetical protein J132_07190 [Termitomyces sp. J132]
Length=804

 Score = 52.0 bits (123),  Expect = 0.003, Method: Compositional matrix adjust.
 Identities = 32/98 (33%), Positives = 43/98 (44%), Gaps = 22/98 (22%)

Query  846  PPIVSSTLSSVPSPKPNPAQNMK------------------KIDPRFPLSPKTSRPVPQI  887
            P + ++TL + PSP P P    K                   IDP         RP+  +
Sbjct  487  PVLSTTTLQTTPSPSPLPIDEKKDERAASDSTPALVVPVMISIDP----VEDPPRPIHTV  542

Query  888  PRVPLDASRLGLYTKDIIKRAWWDACDDMRRCVCSICL  925
            P +P  A+ L  YT D +K  W +AC  + RC CSICL
Sbjct  543  PYIPTTAAHLPPYTMDALKSVWREACAPLYRCRCSICL  580


 Score = 46.6 bits (109),  Expect = 0.17, Method: Compositional matrix adjust.
 Identities = 42/169 (25%), Positives = 72/169 (43%), Gaps = 31/169 (18%)

Query  594  VELTPGGYIQIVEMATEAWIHQRVIDGFWVSPLEQRAFQETFSQQTPSSAEGDITLDVVH  653
            +EL       + +MA   W   R++DG W+SP       +T+   T +  +  +    VH
Sbjct  280  IELLQMEETHVRDMALGDWARARILDGHWMSP------ADTWYHHTVNGYKDYVA--AVH  331

Query  654  PLSSSTLGSSVC----TTSSHP-----NGPLPPAGGALDQKTINLYSRKLRSILLPGLGQ  704
            P   +   SS       T +HP      G +PP  G  +Q   + + R+L+ IL+P +  
Sbjct  332  PCPWTCSPSSDSGHDDETGAHPLPSTLKGTIPPTFGLCEQ-AFSAHQRQLKEILVPPMRN  390

Query  705  IARLCALQ------------KDPLNVAPKLGIHKLL-ELLRLPGAWVHG  740
            + R   ++            +DP   A K+ +  +L EL    G W++G
Sbjct  391  LVRKIVIECQTPGMGYGRGIEDPAVRASKMSVEDVLAELRNEEGVWLNG  439


>gb|KJA20484.1| hypothetical protein HYPSUDRAFT_815010 [Hypholoma sublateritium 
FD-334 SS-4]
Length=953

 Score = 51.6 bits (122),  Expect = 0.004, Method: Compositional matrix adjust.
 Identities = 41/126 (33%), Positives = 57/126 (45%), Gaps = 11/126 (9%)

Query  813  SSHTTSPIRTGSSPPTSLSPPSCAPTIALITPDPPIVSSTLSSVPSPKPNPAQNMKKIDP  872
            SS  TSP+ + ++  T+ SPP           D  + SS   S  +      + M    P
Sbjct  556  SSTATSPVLSTTTLQTTPSPPPLG--------DDGLESSKKDSQLTSTVERKEKMLSSRP  607

Query  873  RF-PLSPKTSRP--VPQIPRVPLDASRLGLYTKDIIKRAWWDACDDMRRCVCSICLRGLK  929
            RF P  P  S P  +  IP VP+  + L  Y+ D IK  W +AC  +  C CSIC R  +
Sbjct  608  RFIPFDPVKSPPRLLGTIPYVPVTVAHLPQYSMDAIKAVWREACSPLYHCSCSICERAKQ  667

Query  930  AQREGR  935
            A+   R
Sbjct  668  AEMIAR  673


>emb|CCA69699.1| hypothetical protein PIIN_03640 [Piriformospora indica DSM 11827]
Length=779

 Score = 50.8 bits (120),  Expect = 0.008, Method: Compositional matrix adjust.
 Identities = 50/191 (26%), Positives = 79/191 (41%), Gaps = 43/191 (23%)

Query  316  LCWVC-HFAR---FPLSQPAAYVRTTLGTVWICKLHLAVPFCGRCLRSRIDPAFRSRRLQ  371
            +C VC  ++R   F L+   +Y     G + ICK H    FCG CL+   D   R     
Sbjct  63   VCAVCVAYSRRLNFALTGATSY-SFAAGEIAICKWHEQHGFCGVCLKD--DTVLREEP--  117

Query  372  WIGPRSSLPEYTAPNEDIDPGLRLTEDDDVEDEDDDINGGVNAGRELYGPNMACCQRCRY  431
                    PEY +    + P         V+DED   +  V             C++CR 
Sbjct  118  --------PEYQS---TLSP---------VDDEDTYFSSLVYT-----------CEQCRT  146

Query  432  ECLQEAVTKRDPAVRIDLEDNGDL-ECFFQYVERAQGSAESIAVDLLERRWLARWTKFHD  490
            +  +  +  R   + + +    D+ E F  Y    +GS +S+ + + ERRWLA  TK  D
Sbjct  147  DACRSQM--RQHKLNLSIAQAVDMDELFSNYQAFGEGSVQSLCIQVQERRWLAVNTKIKD  204

Query  491  LATQVVQTEKI  501
             + Q V  +++
Sbjct  205  FSLQAVAADRL  215


 Score = 48.5 bits (114),  Expect = 0.037, Method: Compositional matrix adjust.
 Identities = 36/122 (30%), Positives = 54/122 (44%), Gaps = 4/122 (3%)

Query  814  SHTTSPIRTGSSPPTSLSPPSCAPTIALITPDPPIVSSTLSSVPSPKPNPAQNMKKIDPR  873
            + T SP  T S+  T+ SPP  A +    +P+    ++T S V     +P  +M    P 
Sbjct  418  TFTESPDGTNSTSKTTPSPPPAAQSKDAKSPEFVAATTTTSDVSKSPRSPIPDM----PE  473

Query  874  FPLSPKTSRPVPQIPRVPLDASRLGLYTKDIIKRAWWDACDDMRRCVCSICLRGLKAQRE  933
              ++   +R V  +P VPL+   L  YT+  I   W  +      C C IC R  + Q E
Sbjct  474  LEVTGVEARQVQSVPFVPLNWGVLPQYTRQWITNLWIKSIHPFVACQCGICKRACQIQEE  533

Query  934  GR  935
             R
Sbjct  534  ER  535


>gb|KDQ56472.1| hypothetical protein JAAARDRAFT_180011 [Jaapia argillacea MUCL 
33604]
Length=865

 Score = 50.8 bits (120),  Expect = 0.008, Method: Compositional matrix adjust.
 Identities = 37/154 (24%), Positives = 71/154 (46%), Gaps = 20/154 (13%)

Query  604  IVEMATEAWIHQRVIDGFWVSPLEQRAFQETFSQQTPSSAEGDITLDVVHPLSSST--LG  661
            + ++A   W   R++DGFW+SP +Q  +    S+++P+       +  +HP   S     
Sbjct  336  VKDLAINDWARNRILDGFWISPADQ-WYNHFDSEKSPA-------IRAIHPCPWSLDPAD  387

Query  662  SSVCTTSSHPNGPLP----PAGGALDQKTINLYSRKLRSILLPGLGQIARLCALQ-----  712
            S+    + HP  P+     P    L +     + R++R++LLP +  I R   ++     
Sbjct  388  SNEPEDAIHPLAPIVRGEIPPSFQLCEFVYRAFQRQMRAVLLPAMTNIVRKLVIECGADG  447

Query  713  KDPLNVAPKLGIHKLLELLRLPGAWVHGGLDCME  746
             DP   A ++ + ++   LR    W + G+D +E
Sbjct  448  VDPAMKALRMDVEEVATALRDDKMW-YNGIDWLE  480


 Score = 43.9 bits (102),  Expect = 0.93, Method: Compositional matrix adjust.
 Identities = 33/103 (32%), Positives = 48/103 (47%), Gaps = 12/103 (12%)

Query  843  TPDPPIVSSTLSSVPSPKPNP--------AQNMKKIDPRFPLSPKTSRP--VPQIPRVPL  892
            T  P + +STL + PSP P+           N     P  P+SP   RP  +  IP VP+
Sbjct  515  TTSPVLSTSTLETTPSPPPSATLKKDGLLGSNGAPAIP-IPVSPVLDRPALLRPIPYVPV  573

Query  893  DASRLGLYTKDIIKRAWWDACDDMRRCVCSICLRG-LKAQREG  934
              S L  ++ +  +  W +AC  + +C C +C R  LKA  E 
Sbjct  574  SISHLPPFSIEAFRLVWREACAPLYQCRCKVCERAMLKANIEA  616


>gb|KIO10281.1| hypothetical protein M404DRAFT_21257 [Pisolithus tinctorius Marx 
270]
Length=1305

 Score = 50.4 bits (119),  Expect = 0.010, Method: Compositional matrix adjust.
 Identities = 72/311 (23%), Positives = 119/311 (38%), Gaps = 77/311 (25%)

Query  677  PAGGALDQKTINLYSRKLRSILLPGLGQIARLCALQK-----------------------  713
            P   +L ++    Y ++LR ILLP +  + R   ++                        
Sbjct  680  PPSFSLCEQAYRAYQKQLRDILLPAMVNLVRKIVVEAMYGVRGWSRFDRDGGCERSSPDG  739

Query  714  ---------DPLNVAPKLGIHKLLELLRLPGAWVHGGLDCMELALDQADGPEQISTMPAE  764
                     DP     ++G+ ++LE LR PG W  G  D + LAL  +      S+ P+E
Sbjct  740  VGRGQRGVVDPAVKVARMGMEEVLEGLRQPGVWFEG-YDWVALALPSSTSSSSPSSSPSE  798

Query  765  ILSPPPLIRHPNNISPSESLSFMPTSSTAKMYTIESPSSTDSISPSAPSSHTTSPIRTGS  824
                           P  +  F     T K    E  +++ +   S  SSHTTSP+ + +
Sbjct  799  ---------------PGGNTKF--GGETTKTEEREEDTNSSTSDRSDTSSHTTSPVLSTT  841

Query  825  SPPTSLSPPSCAPTI------------------ALITPDPPIVSSTLSSV---PSPKPNP  863
            +  T+ SPP     +                  +  T  P +  S  S+     SP    
Sbjct  842  TLQTTPSPPPARGQLEKRGGKVIPPPRVPRPAISTRTTSPSVSGSVTSATGTDASPNTAT  901

Query  864  AQNMKKIDPRF----PLSPKTSRP--VPQIPRVPLDASRLGLYTKDIIKRAWWDACDDMR  917
            A   K   P +    P+SP  +RP  +  IP +P+  + +  ++ +  + AW +AC  + 
Sbjct  902  ATGTKTPKPTYSPPIPISPTLARPRLLHPIPYIPVTLAFMPQFSLEAFRMAWREACAPLF  961

Query  918  RCVCSICLRGL  928
             C CSIC R +
Sbjct  962  HCRCSICERAI  972


 Score = 50.1 bits (118),  Expect = 0.017, Method: Compositional matrix adjust.
 Identities = 49/181 (27%), Positives = 68/181 (38%), Gaps = 36/181 (20%)

Query  206  GGRNGKQWKASDLPLKIIRQIIHLVAISIVTEEVQAKLADGDNRIHFGKRPVGLTIAHIR  265
            G    + W+   LP +++R I   + I + +        D     H   R V  T+    
Sbjct  33   GKHTNRSWQ--HLPPELVRSIATHLLIDLQSSHYTPLTWDAKELWH--PRIVYTTLRDAL  88

Query  266  ARSRIAPLRLVCRAWKQATETIPFWSELATVFGFPHNI----------------------  303
               RI     V  AW  A ET  FWS+  TV   PH++                      
Sbjct  89   DLERIM---QVTPAWAAALETHFFWSKACTVID-PHSVLAQFTILRPKPPPPGSPLPLHH  144

Query  304  -RPYRENDCLITQLCWVCHFARFPLSQPAAYVRTT-----LGTVWICKLHLAVPFCGRCL  357
              PYR    + T+ C+VC       S   A V+ T     LGT+ +C+ H    FCG CL
Sbjct  145  QSPYRHFRQITTRSCYVCRINAPYTSAGLANVKRTFQSLNLGTITLCREHRKSTFCGLCL  204

Query  358  R  358
            R
Sbjct  205  R  205


>gb|KDQ13962.1| hypothetical protein BOTBODRAFT_132833 [Botryobasidium botryosum 
FD-172 SS1]
Length=774

 Score = 49.7 bits (117),  Expect = 0.019, Method: Compositional matrix adjust.
 Identities = 85/348 (24%), Positives = 135/348 (39%), Gaps = 55/348 (16%)

Query  604  IVEMATEAWIHQRVIDGFWVSPLE--QRAFQETFSQQTPSSAEGDITLDVVH-PL-----  655
            I  +A   W   RV+DG W++PL+  Q   Q   + Q   SA  D  +   H PL     
Sbjct  291  IRTIALNDWARNRVLDGCWIAPLDMYQWHLQMQGAIQRGLSAGYDFGIMAAHHPLDLIRD  350

Query  656  --SSSTLGSSVCTTSSHPN-----GPLPPAGGALDQKTINLYSRKLRSILLPGLGQIARL  708
              S   LG +   +S HP+       +PP    +       + + +R++LLP +    R 
Sbjct  351  GESRGRLGRN---SSFHPHPQQIRCAVPPT--RVLHSAQRAFEQNMRTMLLPPMANFVRR  405

Query  709  CALQKDPLNVAPKLGIHKLLELLRLPGAWVHGGLDCMELALDQADGPEQISTMPAEILSP  768
              ++ +  N+         +    L G    GG +C        DG     +      S 
Sbjct  406  LVIECEAGNM-DVCSYASSISFETLIGRHALGGPECW------MDGYNWNFSS-----SV  453

Query  769  PPLIRHPNNISPSESLSFMPTSSTAKMYTIESPSSTDSISPSAPSS--HTTSPIRTGSSP  826
            PP  R  +  + +++             TI S   +D+ +  +PSS  HT+       S 
Sbjct  454  PPRGREDSPSNETDTTDADTDMGHKTESTITSDPHSDTSTRPSPSSTLHTSPSPPPPLSL  513

Query  827  PTSLS-PPSCAPTIALITPDPPIVSSTLS---SVPSPKPNPAQNMKKIDPRFPLSPKTSR  882
            P      PS  PT+     D    S + +   SVPSP   PA  M+ I P          
Sbjct  514  PGDKEISPSAPPTVPSQETDSVTTSKSYTRDASVPSP---PAPAMRVIHP----------  560

Query  883  PVPQIPRVPLDASRLGLYTKDIIKRAWWDACDDMRRCVCSICLRGLKA  930
                IP +P++   +G + + I++  W +A   +  C C ICLR  +A
Sbjct  561  ----IPHLPINPHGVGSFARSIVENLWREATAGLYICQCKICLRTARA  604


 Score = 43.1 bits (100),  Expect = 1.9, Method: Compositional matrix adjust.
 Identities = 44/172 (26%), Positives = 73/172 (42%), Gaps = 22/172 (13%)

Query  344  CKLHLAVPFCGRCLRSRIDPAFRSRRLQWIGPRSSLPEYTAPNEDIDPGLRLTEDDDVED  403
            CK H A  FCG CL+   D   RS    +I P         P+ED   GL L    +V+ 
Sbjct  109  CKAHRARDFCGVCLK---DAVVRS----FIQP--------PPDEDGRYGL-LQCVAEVDA  152

Query  404  EDDDING-GVNAGRELYGPNMACCQRCRYECLQEAVTKRDPAVRIDLEDNGDLECFFQYV  462
            E     G  +N   + +   +A C  CR+  +   +    P +R   ED        ++V
Sbjct  153  EARYAAGIELNEDTQSFPRIVATCTLCRHYAVTNHLRLFAPHIRYQ-EDTHIRRIIDEFV  211

Query  463  ERAQGSAESIAVDLLERRWLARWTKFHDLATQVVQTEKINPVTAASADGNDM  514
            +  +G    +   + E  WL  WT++  L  + V ++++     A ++G D+
Sbjct  212  DFGEGGVRGVMEGIEECIWLKNWTRYEQLWKEAVASQRL----IAYSNGYDV  259


>gb|EKC97870.1| hypothetical protein A1Q2_07873 [Trichosporon asahii var. asahii 
CBS 8904]
Length=890

 Score = 48.1 bits (113),  Expect = 0.055, Method: Compositional matrix adjust.
 Identities = 27/88 (31%), Positives = 42/88 (48%), Gaps = 20/88 (23%)

Query  858  SPKPNPAQNMKKIDPRFPLSPKTSRPVPQI------------------PRVPLDASRLGL  899
            +P+P P Q++K      P +P +   V Q+                  PRVP   S LG 
Sbjct  782  APEPKPTQHIKHAS--TPATPSSGYQVHQMSDSDSTLSSEDFDDFEVAPRVPSATSELGP  839

Query  900  YTKDIIKRAWWDACDDMRRCVCSICLRG  927
              +++I+ AW+ A + +R C CSIC+R 
Sbjct  840  AAEEVIRHAWYRAYEPLRECRCSICVRA  867


>gb|KIL61970.1| hypothetical protein M378DRAFT_25846 [Amanita muscaria Koide 
BX008]
Length=1137

 Score = 48.1 bits (113),  Expect = 0.060, Method: Compositional matrix adjust.
 Identities = 55/231 (24%), Positives = 87/231 (38%), Gaps = 56/231 (24%)

Query  300  PHNIRPYRENDCLITQLCWVCHFARFPLSQPAAYVRTT----------LGTVWICKLH--  347
            P  + PY     ++T  C VC      ++QP +    T          LG + +C+ H  
Sbjct  150  PTRLTPYCHYRNILTYSCHVCR-----VNQPNSSAGLTTAKRLLPSPLLGPIAVCREHDR  204

Query  348  LAVPFCGRCLRSRIDPAFRSRRLQWIGPRSSLPEYTAPNEDIDPGLRLTED---------  398
              + FCG C+R    P F    +         P Y A    I+P   LT           
Sbjct  205  RRIAFCGICMRE--APLFEGTTVP--------PSYVAAG--INPNTALTAQAYNGMSLES  252

Query  399  --DDVEDEDDDINGGVNAGRELYGPNMACCQRCRYECLQEAVTK--RDPAV----RIDLE  450
                +E+ED++   GV           A C+ CR E L    T   RD       ++  E
Sbjct  253  IVSCLENEDEETWPGV----------AATCRFCRREWLWRKATNSARDREAIGGPKLSSE  302

Query  451  DNGDLECFFQYVERAQGSAESIAVDLLERRWLARWTKFHDLATQVVQTEKI  501
            D    +C   +++ A+GS   +     E+ WL ++TK  D+  Q +   + 
Sbjct  303  DWETRQCVDGFIDLAEGSINDVISLAREKWWLRKYTKLGDMMLQALAAARF  353


>gb|KIJ13534.1| hypothetical protein PAXINDRAFT_170416 [Paxillus involutus ATCC 
200175]
Length=1139

 Score = 48.1 bits (113),  Expect = 0.064, Method: Compositional matrix adjust.
 Identities = 61/254 (24%), Positives = 91/254 (36%), Gaps = 67/254 (26%)

Query  301  HNIRPYRENDCLITQLCWVCHF-ARFPLSQPAAYVRT----TLGTVWICKLHLAVPFCGR  355
            + I PYR    +  + C+VC   + F     AA  RT     LG + +C+ H    FCG 
Sbjct  160  YRITPYRHFRQMTGRSCFVCRINSPFSSVGLAAAKRTHNVPNLGPIALCRDHRKTMFCGL  219

Query  356  CLR----SRIDPAF----RSRRL----QWIGPRSSL----PEYTAPNEDIDPGLRLTEDD  399
            CLR    +  DP F     + R     Q  GP S+L      Y      ++P L +    
Sbjct  220  CLREAPPAECDPQFDIHGNTMRFTPYHQPYGPASNLNIGIGAYNPYANVVNPALMVC---  276

Query  400  DVEDEDDDINGGVNAGRELYGPNMACCQRCRYECL----------QEAV-----------  438
              E+ED++   G++            C+ CR ECL          +EAV           
Sbjct  277  CAENEDEETWPGIDT----------TCRACRSECLWQRISISLRDREAVGGPRFIPNSSR  326

Query  439  ------------TKRDPAVRIDLEDNGDLECFFQYVERAQGSAESIAVDLLERRWLARWT  486
                          R P  +    D    +    ++E  +GS   +     E+ WL   T
Sbjct  327  SSSAKDRQVITTVSRYPPAKYASADWETRQTVDAFIELGEGSISEVIAVAREKFWLGSQT  386

Query  487  KFHDLATQVVQTEK  500
            K  +L  Q V   +
Sbjct  387  KMPELMEQAVAATR  400


>gb|KIR70537.1| hypothetical protein I310_05787 [Cryptococcus gattii CA1014]
 gb|KGB79286.2| hypothetical protein CNBG_5124 [Cryptococcus gattii R265]
Length=702

 Score = 47.8 bits (112),  Expect = 0.065, Method: Compositional matrix adjust.
 Identities = 72/327 (22%), Positives = 119/327 (36%), Gaps = 63/327 (19%)

Query  217  DLPLKIIRQIIHLVAISIVTEEVQAKLADGDNRIHFGKRPVGLTIAHIRARSRIAPLRLV  276
            DLP++I+ +I+   A   V  +   +   G N     +R   + +A +R R     +R+V
Sbjct  68   DLPVEILHRILS-YAQDTVPLDFTLQTYYGHN-----QRTREIALALVR-RIWFCRMRMV  120

Query  277  CRAWKQATETIPFWSELATVF-----------------------GFP---HNIRPYREND  310
            C  W+ A ++ PFW E   +                         FP   H  R    + 
Sbjct  121  CTGWRNAVDSHPFWPEFTLLLDPSRHLSSAISGIHSARLAPSTPSFPTLFHRARSSTLSS  180

Query  311  CLITQLCWVCHFARFPLSQPAAYVRTTLGTVWICKLHLAVPFCGRCLRSRIDPAFRS---  367
            CL  +L        +P         +       C+ H A  FC  C+R     A R+   
Sbjct  181  CLACRLNRPSRLGYYPAVGKRLTFTSKFALAPTCEKH-ANNFCSGCMRESEPQAGRAPSP  239

Query  368  --RRLQWIGPRSSLPEYTAPNEDIDPGLRLTEDDDVEDEDDDINGGVNAGRELYGPNMAC  425
                L  I   +   E  +P+E  DP L L++       D D NG     R L  P+   
Sbjct  240  GLHHLTGIEALAESGETRSPSEPADPPLMLSQ---CNHGDVDENGVERFPRSLVCPD---  293

Query  426  CQRCR---------YECLQEAVTKRDPAVRIDLEDNGDLECFFQYVERAQGSAESIAVDL  476
            C++           +EC +    + + ++ +  E   D      Y+    G+A  +    
Sbjct  294  CRKAAIWNEIRLILHECARGGRMRGERSLWLYNEKVKD------YINFNVGTAFEMGYAA  347

Query  477  LERRWL---ARWTKFHDLATQVVQTEK  500
            +E +WL    RW +  + A Q+   EK
Sbjct  348  VEEQWLIDHTRWVELSETALQLQNHEK  374


>gb|KIR32149.1| hypothetical protein I352_05381 [Cryptococcus gattii MMRL2647]
Length=725

 Score = 47.8 bits (112),  Expect = 0.075, Method: Compositional matrix adjust.
 Identities = 72/327 (22%), Positives = 119/327 (36%), Gaps = 63/327 (19%)

Query  217  DLPLKIIRQIIHLVAISIVTEEVQAKLADGDNRIHFGKRPVGLTIAHIRARSRIAPLRLV  276
            DLP++I+ +I+   A   V  +   +   G N     +R   + +A +R R     +R+V
Sbjct  68   DLPVEILHRILS-YAQDAVPLDFTLQTYYGHN-----QRTREIALALVR-RIWFCRMRMV  120

Query  277  CRAWKQATETIPFWSELATVF-----------------------GFP---HNIRPYREND  310
            C  W+ A ++ PFW E   +                         FP   H  R    + 
Sbjct  121  CTGWRNAVDSHPFWPEFTLLLDPSRHHSSAISGIHSAHLAPSTPSFPTLFHRARSSTLSS  180

Query  311  CLITQLCWVCHFARFPLSQPAAYVRTTLGTVWICKLHLAVPFCGRCLRSRIDPAFRS---  367
            CL  +L        +P         +       C+ H A  FC  C+R     A R+   
Sbjct  181  CLACRLNRPSRLGYYPAVGKRLTFTSKFALAPTCEKH-ANNFCSGCMRESEPQAGRAPSP  239

Query  368  --RRLQWIGPRSSLPEYTAPNEDIDPGLRLTEDDDVEDEDDDINGGVNAGRELYGPNMAC  425
                L  I   +   E  +P+E  DP L L++       D D NG     R L  P+   
Sbjct  240  GLHHLTGIEALAESGETRSPSEPADPPLMLSQ---CNHGDVDENGVERFPRSLVCPD---  293

Query  426  CQRCR---------YECLQEAVTKRDPAVRIDLEDNGDLECFFQYVERAQGSAESIAVDL  476
            C++           +EC +    + + ++ +  E   D      Y+    G+A  +    
Sbjct  294  CRKAAIWNEIRLILHECARGGRMRGERSLWLYNEKVKD------YINFNVGTAFEMGYAA  347

Query  477  LERRWL---ARWTKFHDLATQVVQTEK  500
            +E +WL    RW +  + A Q+   EK
Sbjct  348  VEEQWLIDHTRWVELSETALQLQNHEK  374


>ref|XP_007769210.1| hypothetical protein CONPUDRAFT_165798 [Coniophora puteana RWD-64-598 
SS2]
 gb|EIW80204.1| hypothetical protein CONPUDRAFT_165798 [Coniophora puteana RWD-64-598 
SS2]
Length=1289

 Score = 47.8 bits (112),  Expect = 0.081, Method: Compositional matrix adjust.
 Identities = 21/57 (37%), Positives = 32/57 (56%), Gaps = 2/57 (4%)

Query  874  FPLSP--KTSRPVPQIPRVPLDASRLGLYTKDIIKRAWWDACDDMRRCVCSICLRGL  928
             P+SP  ++ +P+P IP VP   +R   Y+ +  K  W +AC  +  C CSIC R +
Sbjct  935  IPISPVLESPKPLPSIPYVPKTLARFAQYSLEAFKIVWREACAPLYHCRCSICERAV  991


>ref|XP_007386122.1| hypothetical protein PUNSTDRAFT_127338 [Punctularia strigosozonata 
HHB-11173 SS5]
 gb|EIN06641.1| hypothetical protein PUNSTDRAFT_127338 [Punctularia strigosozonata 
HHB-11173 SS5]
Length=918

 Score = 47.0 bits (110),  Expect = 0.12, Method: Compositional matrix adjust.
 Identities = 30/96 (31%), Positives = 45/96 (47%), Gaps = 10/96 (10%)

Query  843  TPDPPIVSSTLSSVPSPKPNPAQNMKKIDPRF--------PLSPKTSRPV--PQIPRVPL  892
            T  P + +STL + PSP P   +  +  +           P+SP    PV    IP VP+
Sbjct  525  TTSPVLSTSTLQTTPSPPPAEDKKDEDEEEALRSPATFPIPISPVLESPVLLHAIPYVPV  584

Query  893  DASRLGLYTKDIIKRAWWDACDDMRRCVCSICLRGL  928
             A+ L  Y ++ +K  W +AC  +  C C IC R +
Sbjct  585  SATDLPQYARESLKSIWREACAPLYHCRCRICERAV  620


 Score = 44.7 bits (104),  Expect = 0.61, Method: Compositional matrix adjust.
 Identities = 40/153 (26%), Positives = 65/153 (42%), Gaps = 31/153 (20%)

Query  604  IVEMATEAWIHQRVIDGFWVSPLEQRAFQETFSQQTPSS-----AEGDITL------DVV  652
            I  +A +AW  +RV+DG+W SP +Q  +     +  P +     A   I+       D  
Sbjct  343  IKMLAIDAWARRRVLDGYWCSPADQ--WYNMIPRDKPVTPAEHPASWSISYDSEDDPDAP  400

Query  653  HPLSSSTLGSSVCTTSSHPNGPLPPAGGALDQKTINLYSRKLRSILLPGLGQIARLCALQ  712
            HPL   T+G              PP  G  +Q     + R+LR++LLP +  I R   ++
Sbjct  401  HPL-PETVGVET-----------PPTYGLCEQAH-GAWQRQLRNVLLPAMNNIVRRLVIE  447

Query  713  -----KDPLNVAPKLGIHKLLELLRLPGAWVHG  740
                  D    A K+ + +++  LR    W +G
Sbjct  448  CTADGVDAALKASKMTLDEVVAELRREETWFNG  480


>ref|XP_001881514.1| ABC domain-containing protein [Laccaria bicolor S238N-H82]
 gb|EDR07725.1| ABC domain-containing protein [Laccaria bicolor S238N-H82]
Length=963

 Score = 45.4 bits (106),  Expect = 0.38, Method: Compositional matrix adjust.
 Identities = 39/166 (23%), Positives = 73/166 (44%), Gaps = 38/166 (23%)

Query  604  IVEMATEAWIHQRVIDGFWVSPLEQRAFQETFSQQTPSSAEGDITLDVVHP--------L  655
            + ++A   W   R++DG+W SP +   +Q +   Q          +  +HP        L
Sbjct  460  VRDLALGDWARARILDGYWFSPADI-WYQYSVPGQP-------AVVPAIHPCPWTRERSL  511

Query  656  SSSTLGSSVCTT-------SSHPN-----GPLPPAGGALDQKTINLYSRKLRSILLPGLG  703
            S+++ GS+V TT         HP        +PP+    +Q  +  + R++R +LLP L 
Sbjct  512  SNASSGSAVLTTDEAEEVEEEHPKRSTFTAEIPPSYSLCEQAYV-AHQRQMRVVLLPVLK  570

Query  704  QIARLCALQ--------KDPLNVAPKLGIHKLLELLR-LPGAWVHG  740
             + R   ++        +DP   A ++ + ++L ++R   G W  G
Sbjct  571  NVVRRLVMECAASEGRREDPAVRAARMNMEEVLRIVREEEGVWFDG  616


>ref|XP_007850649.1| abc domain-containing protein [Moniliophthora roreri MCA 2997]
 gb|ESK90033.1| abc domain-containing protein [Moniliophthora roreri MCA 2997]
Length=886

 Score = 45.1 bits (105),  Expect = 0.45, Method: Compositional matrix adjust.
 Identities = 29/86 (34%), Positives = 43/86 (50%), Gaps = 5/86 (6%)

Query  846  PPIVSSTLSSVPSPKPN---PAQNMKKIDPRFPLSPKTSRP--VPQIPRVPLDASRLGLY  900
            P + +STL + PSP P+     Q  K+      + P  + P  +  IP VPL A+ L  +
Sbjct  508  PVLSTSTLQTTPSPPPSTDGKDQTAKRRPVTIAVDPVLNPPRLLHPIPYVPLMAAHLPQF  567

Query  901  TKDIIKRAWWDACDDMRRCVCSICLR  926
            + +  K  W +AC  +  C CSIC R
Sbjct  568  SMEAFKGVWREACQPLYHCRCSICER  593


>gb|EPS94938.1| hypothetical protein FOMPIDRAFT_1169167 [Fomitopsis pinicola 
FP-58527 SS1]
Length=776

 Score = 44.7 bits (104),  Expect = 0.55, Method: Compositional matrix adjust.
 Identities = 29/89 (33%), Positives = 46/89 (52%), Gaps = 6/89 (7%)

Query  846  PPIVSSTLSSVPSPKPNPAQN---MKKIDPRFPLSPKTSRP---VPQIPRVPLDASRLGL  899
            P + ++TL + PSP P+  ++   M       P + +TSR    +  IP VP   S +  
Sbjct  522  PGLSTTTLQTTPSPPPSSVKDDEGMSSPVSALPPALETSREAHFMHPIPYVPSKLSHMPE  581

Query  900  YTKDIIKRAWWDACDDMRRCVCSICLRGL  928
            YT  +++ AW  AC  + +C CSIC R +
Sbjct  582  YTIGMLRSAWACACAPVYQCYCSICRRRM  610


>gb|KIK25902.1| hypothetical protein PISMIDRAFT_28753 [Pisolithus microcarpus 
441]
Length=1299

 Score = 44.7 bits (104),  Expect = 0.64, Method: Compositional matrix adjust.
 Identities = 33/113 (29%), Positives = 43/113 (38%), Gaps = 27/113 (24%)

Query  273  LRLVCRAWKQATETIPFWSELATVF-------------------GFP---HNIRPYREND  310
            L  V  AW  A ET  FW++  TV                    G P   H+  PYR   
Sbjct  91   LMQVTPAWAAALETHLFWAKACTVIDPHAVLAHFSILRSKPSQPGAPLPLHHQTPYRHFR  150

Query  311  CLITQLCWVCHFARFPLSQPAAYVRTT-----LGTVWICKLHLAVPFCGRCLR  358
             +  + C+VC       S      + T     LGT+ +C+ H    FCG CLR
Sbjct  151  QMTARSCYVCRINAPYTSAGLTNAKRTIEALNLGTIALCREHRKSTFCGLCLR  203


 Score = 42.4 bits (98),  Expect = 3.2, Method: Compositional matrix adjust.
 Identities = 19/59 (32%), Positives = 32/59 (54%), Gaps = 2/59 (3%)

Query  872  PRFPLSPKTSRP--VPQIPRVPLDASRLGLYTKDIIKRAWWDACDDMRRCVCSICLRGL  928
            P  P+SP  +RP  +  IP +P+  + +  ++ +  K  W +AC  +  C CSIC R +
Sbjct  916  PPIPISPTLARPRLLHPIPYIPITLAYMPQFSLEAFKVVWREACAPLFHCRCSICERAI  974


>ref|XP_007866498.1| hypothetical protein GLOTRDRAFT_121520 [Gloeophyllum trabeum 
ATCC 11539]
 gb|EPQ55366.1| hypothetical protein GLOTRDRAFT_121520 [Gloeophyllum trabeum 
ATCC 11539]
Length=820

 Score = 44.3 bits (103),  Expect = 0.81, Method: Compositional matrix adjust.
 Identities = 30/116 (26%), Positives = 56/116 (48%), Gaps = 25/116 (22%)

Query  815  HTTSPIRTGSSPPTSLSPPSCAPTIALITPDPPIVSSTLSSVPSPKPNPAQNMKKIDPRF  874
            H TSP+ + S+  T+ SP             PP+V+         K +  + ++++    
Sbjct  480  HATSPVLSTSTLGTTPSP-------------PPLVTK--------KDDEVEGLRQVP--I  516

Query  875  PLSP--KTSRPVPQIPRVPLDASRLGLYTKDIIKRAWWDACDDMRRCVCSICLRGL  928
            P+SP  +T R +  IP +P+  S +  ++++  K  W +AC  + +C C IC R +
Sbjct  517  PVSPVLETPRLLHPIPYIPVTTSHMPPFSQEAFKMVWREACQPLYQCRCKICERAM  572


>gb|KIM55442.1| hypothetical protein SCLCIDRAFT_1221129 [Scleroderma citrinum 
Foug A]
Length=1170

 Score = 44.3 bits (103),  Expect = 0.87, Method: Compositional matrix adjust.
 Identities = 112/508 (22%), Positives = 177/508 (35%), Gaps = 129/508 (25%)

Query  206  GGRNGKQWKASDLPLKIIRQIIHLVAISIVTEEVQAKLADGDNRIHFGKRPVGLTIAHIR  265
            G +  + W  ++LP +IIR I   + + +          D     H       +T   +R
Sbjct  13   GKKTHRSW--NNLPPEIIRTIATFLLLDVQLSNYCPLSWDAAELWH-----PRITYTTLR  65

Query  266  ARSRIAPLRLVCRAWKQATETIPFWSELATVFGFPHNI----------------------  303
                I  L  V   W  A ET  FW++  TV   PH+I                      
Sbjct  66   DALDIEKLMQVSPLWAVALETHLFWTKACTVID-PHDILAHHAIVRASAPVTSTGTAGAA  124

Query  304  -----------RPYRENDCLITQLCWVCHFAR-FPLSQPAAYVRT----TLGTVWICKLH  347
                        PYR    L ++ C VC     +  +  AA  RT     LG V +C+ H
Sbjct  125  TTTAQSQHFRITPYRHFRLLTSRSCIVCRINTPYSSTGLAAAKRTLPVPVLGNVTLCRDH  184

Query  348  LAVPFCGRCLR----SRIDPAF--RSRRLQWIGPRS----SLPEYTAPNEDIDPGLRLTE  397
                FCG CLR    +  DP     +  L++ G +        +  AP    +P L +  
Sbjct  185  RKSMFCGLCLREAPSAEYDPPLDIHANTLRFTGYQQHHAHGYQQQYAPIS-FNPALMVC-  242

Query  398  DDDVEDEDDDINGGVNAGRELYGPNMACCQRCRYECLQEAVTK--RD-----------PA  444
                E+EDDD   GV             C+ CR E L + ++   RD           P+
Sbjct  243  --CAENEDDDTWPGVET----------TCRSCRCEYLWQRISTSMRDREAIGGPRFVPPS  290

Query  445  VRIDLEDNGDLECFFQ----------------------YVERAQGSAESIAVDLLERRWL  482
                   +G +E  F                       +++  +GS   +     E+ WL
Sbjct  291  TTSPFTPSGVIEGTFATSRYPPAKYASSDWEIRQTVDAFIDLGEGSISEVIAVAREKFWL  350

Query  483  ARWTKFHDLATQVVQTEKINPVTA--ASADGNDMRAIFKASMGIEGDGSEPMTEALEREL  540
               TK  ++  Q V   +    T+  A+A     R+ +              +E +   +
Sbjct  351  RSHTKMTEIMEQAVAATRWTGETSSLATASAACARSPYS-------------SEYVSNRV  397

Query  541  AQFEYEAAVAREENAARVVGAEHNNSDGERQTDEEGQGQEDEEEEEEEEEEEGVELTPGG  600
             +   EAA   E N A  V  E    + E + +EE + +E+E+ E     E         
Sbjct  398  VEGP-EAAANDEGNDAAAVEEEGEEEEEEEEEEEEEEEEEEEDPELLSLTET--------  448

Query  601  YIQIVEMATEAWIHQRVIDGFWVSPLEQ  628
               + ++A   W   R++DG W SP +Q
Sbjct  449  CSTVRDLAVSDWARTRILDGHWCSPADQ  476


 Score = 42.4 bits (98),  Expect = 3.7, Method: Compositional matrix adjust.
 Identities = 29/104 (28%), Positives = 46/104 (44%), Gaps = 22/104 (21%)

Query  846  PPIVSSTLSSVPSPKP-----NPAQNMKKID--------------PR--FPLSPKTSRPV  884
            P + ++TL + PSP P         N+ + D              P+  F   PKT R +
Sbjct  686  PVLSTTTLHTTPSPPPVRGARAGGDNLAEHDGDRMVEQKDDQEGEPKETFEQRPKT-RLI  744

Query  885  PQIPRVPLDASRLGLYTKDIIKRAWWDACDDMRRCVCSICLRGL  928
              IP +P+  + +  Y+ +  K  W DAC  +  C C+IC R +
Sbjct  745  HPIPYIPVTIAHMAQYSLEAFKSVWRDACAPLFHCQCTICERAV  788


>gb|KIR52971.1| hypothetical protein I315_04429 [Cryptococcus gattii Ru294]
Length=1029

 Score = 43.9 bits (102),  Expect = 1.1, Method: Compositional matrix adjust.
 Identities = 55/261 (21%), Positives = 86/261 (33%), Gaps = 48/261 (18%)

Query  273  LRLVCRAWKQATETIPFWSELATVF--------------------GFPHNIRPYRENDCL  312
            +R+VC  W+ A ++ PFW E   +                        H  R      CL
Sbjct  1    MRMVCTGWRHAVDSHPFWPEFTLLLDPSRHHSSAISTTPPPPPFPTLFHRARSSTLASCL  60

Query  313  ITQLCWVCHFARFPLSQPAAYVRTTLGTVWICKLHLAVPFCGRCLRSRIDPAFRSRRLQW  372
              +L        +P  +      +       C+ H +  FC  C+R     A R+     
Sbjct  61   ACRLNRPSRLGYYPAVRKRLTFTSKFALAPTCEKH-SNNFCSGCMRESEPQAGRAPS-PG  118

Query  373  IGPRSSLPEYTAPNEDIDPGLRLTEDDDVEDEDDDINGGVNAGRELYGPNMACCQRCRYE  432
            IG      E  +P+E   P L L++       D D NG     R L  P+          
Sbjct  119  IGALVEAGETRSPSEPAVPALMLSQ---CNHGDVDENGVERFPRSLVCPD----------  165

Query  433  CLQEAVTKRDPAVRIDLEDNGDL----------ECFFQYVERAQGSAESIAVDLLERRWL  482
            C + A+      +  D    G +          E    Y+    G+A  +    +E +WL
Sbjct  166  CRKAAIWNEIRLILHDCARGGRMRGERSLWLYNEKVKDYINFNVGTAFEMGYAAVEEQWL  225

Query  483  ---ARWTKFHDLATQVVQTEK  500
                RW +  + A Q+   EK
Sbjct  226  IDHTRWVELSETALQLQNHEK  246


>ref|XP_009553209.1| hypothetical protein HETIRDRAFT_461425 [Heterobasidion irregulare 
TC 32-1]
 gb|ETW74721.1| hypothetical protein HETIRDRAFT_461425 [Heterobasidion irregulare 
TC 32-1]
Length=819

 Score = 43.5 bits (101),  Expect = 1.3, Method: Compositional matrix adjust.
 Identities = 34/143 (24%), Positives = 62/143 (43%), Gaps = 22/143 (15%)

Query  603  QIVEMATEAWIHQRVIDGFWVSPLEQRAFQETFSQQTPSSAEGDITLDVVHPLSSSTLGS  662
            Q+  MA   W   R++DG+WVSP +       +    P+S      +  +HP+  S    
Sbjct  328  QVRVMAVNDWARVRILDGYWVSPADD------WYNNRPAS------VSTMHPIPWSLDDE  375

Query  663  SVCTTSSHPNG-----PLPPAGGALDQKTINLYSRKLRSILLPGLGQIARLCALQ--KDP  715
               +  +HP       P  P     D      + R++R ++LP +  IA+   ++   DP
Sbjct  376  R--SGQTHPTARTLRMPAAPTFTLAD-TLYRAFQRQMRILVLPAMMNIAKRIEMEGRGDP  432

Query  716  LNVAPKLGIHKLLELLRLPGAWV  738
            +    ++G+ ++L  L+    WV
Sbjct  433  VVRVSRMGMDEVLRTLQQGWPWV  455


>gb|KIK82139.1| hypothetical protein PAXRUDRAFT_832381 [Paxillus rubicundulus 
Ve08.2h10]
Length=1163

 Score = 42.7 bits (99),  Expect = 2.6, Method: Compositional matrix adjust.
 Identities = 44/178 (25%), Positives = 72/178 (40%), Gaps = 42/178 (24%)

Query  604  IVEMATEAWIHQRVIDGFWVSPLEQR-------------AFQETFSQQTPS-------SA  643
            + E+A   W   R++DG W SP +Q              A +ET S+  P+       + 
Sbjct  500  VRELAISDWARTRILDGHWCSPADQWYGYAWPAGGGVRDAGEETESEPDPNEELREGCTV  559

Query  644  EGDITLDVVHPLSSSTLGSSVCTTSS----------HP-----NGPLPPAGGALDQKTIN  688
                 ++ VHP   +   SS   +            HP        +PP+  AL ++   
Sbjct  560  RRTTRVEAVHPCPLTISSSSPSFSLPSSPTSEMDDMHPRSSTVRAQVPPS-FALCEQAYR  618

Query  689  LYSRKLRSILLPGLGQIARLCALQK------DPLNVAPKLGIHKLLELLRLPGAWVHG  740
             Y ++LR ILLP +  + R   ++       DP   A ++ + ++L  LR PG W  G
Sbjct  619  AYQKQLRLILLPPMNNLVRKFVIEYSSGIAGDPAVRAARMELEEVLAELRAPGVWFDG  676


>ref|XP_005383497.1| PREDICTED: receptor-transporting protein 3 [Chinchilla lanigera]
 ref|XP_005383498.1| PREDICTED: receptor-transporting protein 3 [Chinchilla lanigera]
 ref|XP_005383499.1| PREDICTED: receptor-transporting protein 3 [Chinchilla lanigera]
 ref|XP_013369043.1| PREDICTED: receptor-transporting protein 3 [Chinchilla lanigera]
 ref|XP_013369044.1| PREDICTED: receptor-transporting protein 3 [Chinchilla lanigera]
Length=472

 Score = 42.0 bits (97),  Expect = 3.3, Method: Compositional matrix adjust.
 Identities = 46/159 (29%), Positives = 68/159 (43%), Gaps = 26/159 (16%)

Query  758  ISTMPAEILSPPPLIRHPNNISPSESLSFMPTSSTAKMYTIESPSSTDSISPSAPSSHTT  817
            +ST+ A+ L+PP              +S  P  S A  Y  E  S   +++P AP    T
Sbjct  211  VSTVTAKDLNPP---------KAESKVSHTPKPSAAPRYPTEEVSPGLTVTPEAPHPLKT  261

Query  818  SP-IRTGSSPPTSLSPPS--CAPTIALIT--PDPPIVSSTLSSVPSPKPNPAQNMKKIDP  872
             P +   S P ++L  P+    PT+ L    P+PP   S +S  P P   P    +++ P
Sbjct  262  DPKVSQASKPSSTLKIPTKEVLPTLTLTPKFPNPPKAESKVSHTPKPSAVPRYPTEEVSP  321

Query  873  RFPLSPKTSRPV---PQI---------PRVPLDASRLGL  899
            R  L+PK   P    P++         PR P +   LGL
Sbjct  322  RLTLTPKCPNPPKADPKVSHAPKPSAAPRYPTEEESLGL  360


Lambda      K        H        a         alpha
   0.315    0.132    0.395    0.792     4.96 

Gapped
Lambda      K        H        a         alpha    sigma
   0.267   0.0410    0.140     1.90     42.6     43.6 

Effective search space used: 11961422019760


  Database: nr
    Posted date:  Sep 23, 2015 12:05 AM
  Number of letters in database: 26,053,659,533
  Number of sequences in database:  71,551,133


Matrix: BLOSUM62
Gap Penalties: Existence: 11, Extension: 1
Neighboring words threshold: 11
Window for multiple hits: 40
```
